# Design, Synthesis, and Evaluation of Small Fluorescent Molecules with a 1,1-Dimethylnaphthalen-2-(1*H*)-One Core

**DOI:** 10.3390/molecules29143396

**Published:** 2024-07-19

**Authors:** Zhengyang Wang, Shuting Wang, Yuexing Zhang, Mingliang Ma

**Affiliations:** 1 Shanghai Engineering Research Center of Molecular Therapeutics and New Drug Development, School of Chemistry and Molecular Engineering, East China Normal University, Shanghai 200062, China; 2 Shandong Provincial Key Laboratory of Monocrystalline Silicon Semiconductor Materials and Technology, Shandong Universities Engineering Research Center of Integrated Circuits Functional Materials and Expanded Applications, College of Chemistry and Chemical Engineering, Dezhou University, Dezhou 253023, China

**Keywords:** fluorescence, near-infrared, small molecule, visualization

## Abstract

A series of fluorescent molecules with 1,1-dimethylnaphthalene-2(1*H*)-one as the core were synthesized to overcome aggregation quenching and emit bright green fluorescence. The low molecular weight of these molecules led to them to smoothly pass through the cell membrane and penetrate deep into the nucleus to emit the corresponding fluorescence. Among them, NC-4-Br and NC-5-3O have good optical and in vitro properties and showed potential for use as fluorescent probes.

## 1. Introduction

Fluorescence imaging has many practical applications in chemistry, biology, clinical medicine, and other fields [1,2]. Generally, it can be divided into photoluminescence (PL), thermoluminescence (TL), and mechanoluminescence (ML) [3,4,5,6]. Among them, donor–acceptor (D-A)-type molecules have attracted much attention. Due to the unique characteristics of electrons, the construction of D-A-conjugated light-emitting structures has propelled the advancement of fluorescent small molecules [7,8,9,10]. Electrons transition freely in the entire conjugation interval within the D-A-D structure. In this structure, the effective conjugation area expands as the energy band gap decreases, which changes the fluorescence characteristics of the target compounds [11,12,13,14]. By modifying the conjugated skeleton or terminal substituents, different fluorescent molecules with specific application requirements can thus be achieved [15,16].

In practical applications, fluorescence in the near-infrared region is obvious with no additional organic solvent. The fluorescence excitation–emission spectrum can be obtained within 20 s, providing measurements without damaging the properties of the sample itself. In addition, the visual detection results can be directly obtained without destroying the growth of tissues or cells [17,18]. Therefore, fluorescent small molecules are relatively safe with good applicability.

**Figure 1:**
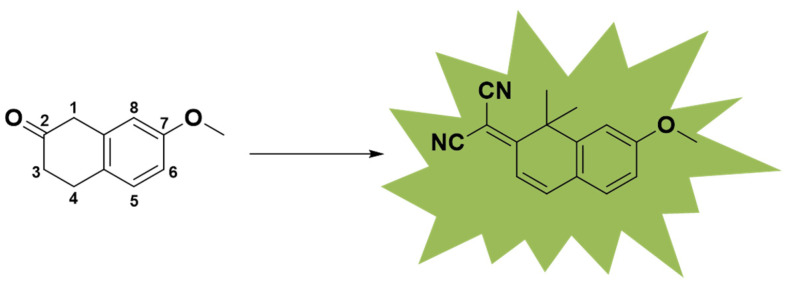
Fluorescent core of small-molecule NC compounds.

In a previous work, a series of small molecules with fluorescence properties were synthesized. However, these molecules could not overcome aggregation quenching in water. In this work, we focused on the 1,1-dimethylnaphthalene-2(1*H*)-one nucleus, which has been reported previously (Figure 1) [19]. To obtain molecules that can be excited at UV–visible wavelengths and emit in the near-infrared region, we used a transformational form of coumarin as the core. Utilizing the excellent cell permeability of coumarin and introducing difluoromethylene substituents into the structure, a hydrophobic environment can be built [20].

**Scheme 1:**
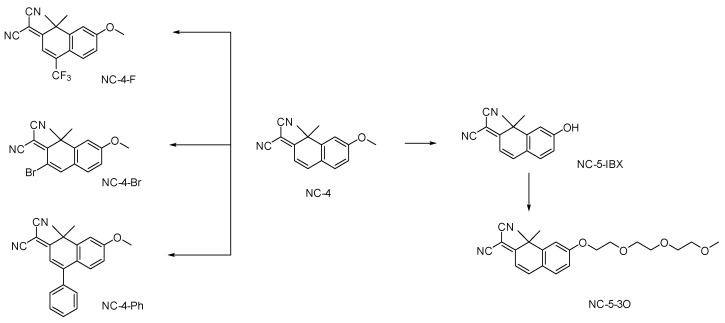
Design and synthesis of small-molecule NC compounds.

For the NC (nitrogen coumarin) compounds, we chose 1,1-dimethylnaphthalene-2(1*H*)-one as the core nucleus [21]. To fully enhance the fluorescence emission characteristics of these compounds and expand the conjugated region, we referred to the fluorescent molecule library constructed by Martin J [19]. As reported, a dimethyl group was introduced into the 1-position of the parent nucleus, and the electron-donating effect of σ-π hyperconjugation was enhanced [22]. The malononitrile group is a strong electron acceptor and was introduced at the 7-position, leading to the lone electron pair of N providing a p–π hyperconjugation effect [23,24]. The electron-withdrawing and electron-donating parts of the molecular structure were connected by a carbon–carbon double bond. In order to form a local D–π–A structure, a carbon–carbon double bond was introduced as a bridge for transition communication by connecting the local structure with the large π-conjugated fluorophore of the benzene ring (Scheme 1, Figures S1 and S2).

## 2. Results and Discussion

### 2.1. Spectral Properties

**Figure 2:**
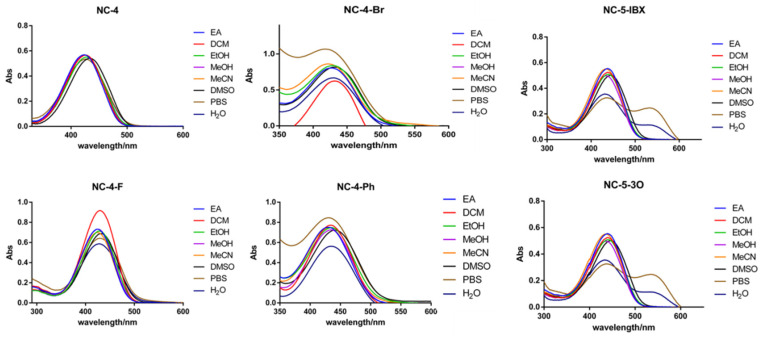
UV absorption spectra of 50 μM compounds in different solvents.

The NC compounds were tested using ultraviolet absorption and fluorescence excitation–emission spectrometers. Eight organic solvents and aqueous solvents with different polarities were introduced, and the polarities were ranked as EA < DCM < EtOH < MeOH < MeCN < DMSO < PBS < H_2_O. As shown in the ultraviolet absorption spectrum of each compound, the ultraviolet absorption wavelengths of the NC-4 compounds were in a range of 420–440 nm, which is in the visible light region (Figure 2). In addition, the NC-5 compounds were expanded with a polyethyleneglycol (PEG) chain based on the structure of NC-4 without modifying the central conjugated structure and had similar excitation energies. In PBS buffer and ultrapure water, the NC-5 (Figures S4 and S5; Tables S4 and S5) compounds were absorbed at 400–500 nm with a double-headed peak waveform, and the absorption intensity was slightly lower than in the organic solvent. The hydroxyl and PEG chains are related and discussed in detail in the frontier orbital calculations.

**Figure 3:**
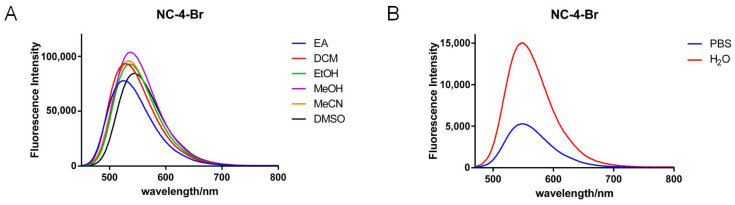
Fluorescence emission spectra of NC-4-Br in different solvents. (**A**) Fluorescence emission spectra of NC-4-Br in organic solvent; (**B**) Fluorescence emission spectra of NC-4-Br in PBS and water.

The wavelength interval of the Stokes shift for the NC-4 compounds in ultrapure water (Figure S1) was 100–150 nm, which is a relatively moderate excitation–emission wavelength interval with no interference (Figure 3).

In each solvent, the fluorescence intensity increased nearly 2–4-fold, indicating that the greater polarity of water did not affect the fluorescence emission of the NC-4 compounds (Table S1). Notably, NC-4-Ph has a fluorescence intensity nearly one order of magnitude lower than that of other compounds (Figure S3; Table S3). This may be due to the connection of the benzene ring at the 4-position of the coumarin core. When the polarity of the molecule is reduced, the rotation of the single bond increases molecular steric hindrance and the uneven distribution of the electron cloud. This improves the overall conjugation of the compound but decreases its overall fluorescence emission characteristics. In addition, the emission wavelengths of NC-4-F (Figure S2, Table S2) and NC-4-Br were close to 600 nm (Table 1), which is promising for near-infrared fluorescent small molecules.

### 2.2. Calculation of the Electron Transition Orbital Energy of NC Compounds

**Figure 4:**
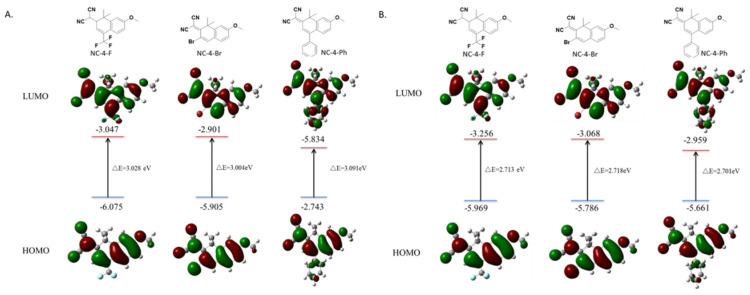
Frontier orbital distributions, energy levels calculated by Gaussian 16 in the ground state and first excited state: (**A**) ground state; (**B**) first excited state.

Based on the characterization data of the UV fluorescence properties, the frontier orbits of all compounds were measured, and the energy required by π electrons in the structure of each compound during the level transition was calculated by the time-dependent density functional theory. Calculations using the Gaussian 16, Revision A.03 software showed that, to generate a fluorescence emission, NC-4-F, NC-4-Br, and NC-4-Ph, respectively, absorbed 3.028, 3.004, and 3.091 eV of energy and released 2.713, 2.718, and 2.701 eV. These derivative compounds connect electron-withdrawing groups depending on the fluorescent parent nucleus. Only slight differences in the energy absorption and release values were observed, which were all in the normal range (2.2–3.6 eV) of the electron delocalization of fluorescent small molecules. It is worth mentioning that, in the molecular model of NC-4-Ph, the benzene ring at the 4-position of the ground state or the first excited state can never be in the same plane as the two six-membered rings of the central structure (Figure 4). This is likely due to the steric hindrance of the benzene ring being much larger than that of the F or Br atoms, which hinders the formation of an integrated large π delocalized region, which may also account for the lower fluorescence intensity of NC-4-Ph compared with other compounds (Figure 4).

After NC-5 was connected to the PEG chain (Figure S6), the ground state absorption energy of NC-4-3O was almost the same as that of NC-5-IBX (about 3.2 eV), indicating that the length of the PEG chain affected the water solubility of the compound with little effect on the fluorescence properties of the molecules.

### 2.3. Viscosity Sensitivities of the NC Compounds

To further explore whether the NC compounds can effectively enter the cell interior, an artificial viscous environment was created to test the viscosity dependence. A viscosity gradient using mixed solvents of PEG400 and ultrapure water was used to simulate dyeing and luminescence after passing through the cell membrane and entering the interior of the cell.

**Figure 5:**
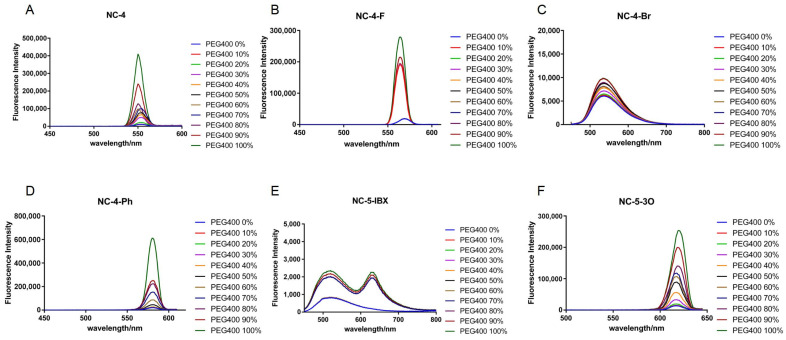
Fluorescence emission spectra of compounds at different viscosities. (**A**) Fluorescence emission spectra of compound NC-4 at PEG400 from 0-100% viscosity gradient; (**B**) Fluorescence emission spectra of compound NC-4-F at PEG400 from 0-100% viscosity gradient; (**C**) Fluorescence emission spectra of compound NC-4-Br at PEG400 from 0-100% viscosity gradient; (**D**) Fluorescence emission spectra of compound NC-4-Ph at PEG400 from 0-100% viscosity gradient; (**E**) Fluorescence emission spectra of compound NC-5-IBX at PEG400 from 0-100% viscosity gradient; (**F**) Fluorescence emission spectra of compound NC-5-3O at PEG400 from 0-100% viscosity gradient.

Under an excitation wavelength of around 430 nm, all NC-4 compounds showed a corresponding gradient in fluorescence intensity as the ambient viscosity increased, but the magnitude was tiny (Figure 5A–D). In addition, it can be seen from the fluorescence emission wavelength that the NC-4 compounds have fluorescence emission characteristics of around 550 nm, which were slightly red-shifted compared with the ultrapure water, and that they shifted in the long-wave direction by about 5–10 nm (Figure 5A–D). These derivatives all have maximum fluorescence emissions near 600 nm. Due to the higher concentrations of compounds used in the experiments than in previous fluorescence spectroscopic tests, the fluorescence intensities were comparatively exponentially enhanced in the presence of viscous solvents (Figure 5).

The NC-5 compounds showed an enhanced fluorescence intensity in a viscous environment with a more obvious gradient change than those of the NC-4 compounds (Figure 5E,F). The presence of water molecules provides the solvent with a certain viscosity, causes the PEG chain to not easily entangle, maintains the energy levels between the molecules, and allows the electrons to effectively absorb and release energy within the molecules. The effect of a viscous solvent on NC-5-IBX is different than for other molecules. NC-5-IBX had a single fluorescence emission peak in ultrapure water but showed no change when PEG400 was added to the solvent.

In short, viscosity had no essential effect on the above two compounds and even contributed to stronger fluorescence emissions. The viscosity tests show that these compounds can effectively pass through the cell membrane and can effectively generate fluorescence in an aqueous environment.

### 2.4. Fluorescence Lifetimes of NC Compounds

**Figure 6:**
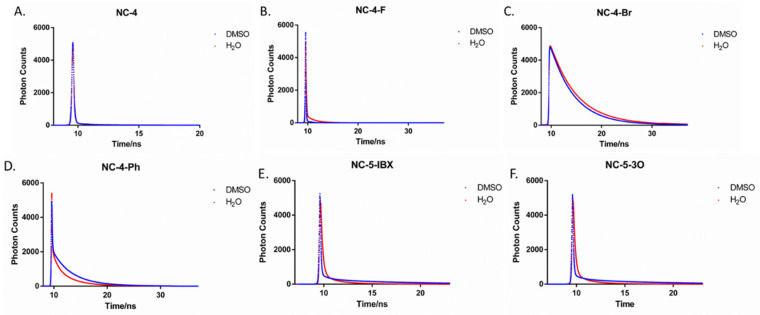
Fluorescence decay of compounds in DMSO and H_2_O. (**A**) Fluorescence lifetime spectra of compound NC-4 in DMSO and H_2_O; (**B**) Fluorescence lifetime spectra of compound NC-4-F in DMSO and H_2_O; (**C**) Fluorescence lifetime spectra of compound NC-4-Br in DMSO and H_2_O; (**D**) Fluorescence lifetime spectra of compound NC-4-Ph in DMSO and H_2_O; (**E**) Fluorescence lifetime spectra of compound NC-5-IBX in DMSO and H_2_O; (**F**) Fluorescence lifetime spectra of compound NC-5-3O in DMSO and H_2_O.

The fluorescence lifetimes of NC-4 compounds vary greatly, with NC-4-Br being the most stable (Figure 6). In DMSO, the lifetime of NC-4 was only 0.49 ns, and the average lifetime of NC-4-F was 1.18 ns (Table 2), both of which are more easily quenched compounds. NC-3-Br had the longest fluorescence lifetime of up to 4.64 ns, which means that NC-4-Br can also maintain its structure under high-energy excitation. As can be seen from Table 2, the NC-5 compounds generally have a slightly longer fluorescence lifetime in ultrapure water than in the organic phase. NC-5-IBX had the longest lifetime (2.06 ns) compared with the other compounds, while the PEG chain linked to NC-5-3O seemed to weaken its fluorescence stability.

Notably, the fluorescence lifetime of NC-4-Br in ultrapure water is close to 6 ns (Table 2), which is nearly 9-fold that of NC-4 (Figure 6). As shown by the experimental results, NC-4-Br had the highest fluorescence stability, exhibiting an excellent fluorescence lifetime length and optical stability in both the organic and aqueous phases.

### 2.5. Biological Evaluation

**Figure 7:**
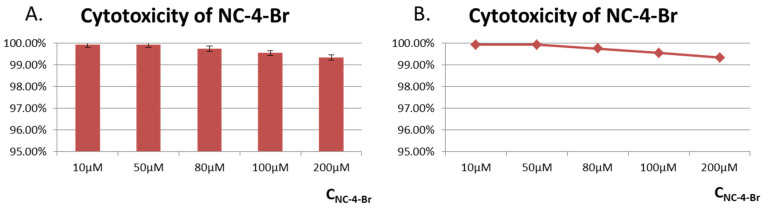
Cytotoxicity of NC-4-Br in GES-1 cells. (**A**) Toxicity evaluation histogram of compound NC-4-Br in GES-1 cells; (**B**) Line diagram of toxicity evaluation of compound NC-4-Br in GES-1 cells.

GES-1 cells, known as healthy human gastric parietal cells, were used for cytotoxicity assays. GES-1 cells were treated with different concentrations (10–200 M) of NC compounds (Figure 7 and Figures S7–S11). CCK-8 assays showed that all cells exhibited high cell viabilities of over 96%, indicating the low toxicity of NC compounds toward healthy cells and their great potential for the creation of fluorescent probes.

**Figure 8:**
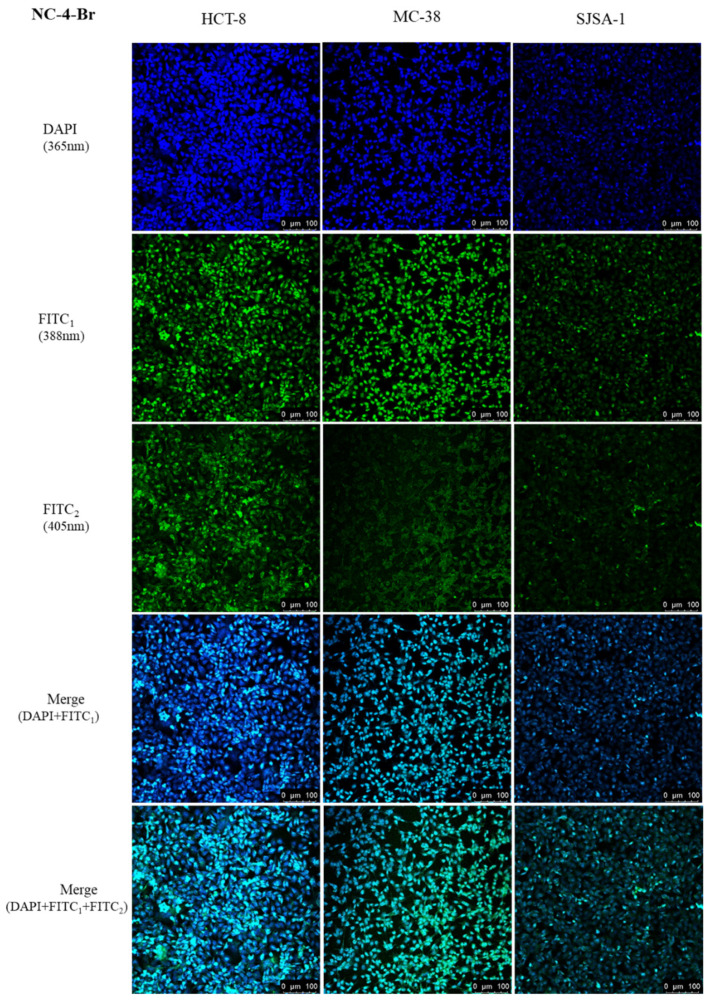
In vitro cellular uptake of NC-4-Br in three cancer cell lines.

Based on the above results, we evaluated the tumor bioimaging ability of the NC compounds in HCT-8, MC-38, and SJSA-1 cell lines by using single-photon laser confocal microscopy. The cells were incubated at an appropriate density with 50 μM of NC compounds for 24 h at 37 °C and 5% CO_2_. As shown in Figure 8, strong fluorescence was observed in the cytoplasm of all groups with tumor-selective bioimaging capabilities.

To further confirm the subcellular distribution of NC compounds following their uptake into tumor cells, 4′,6-diamidino-2-phenylindole was used to selectively and strongly stain the intact nuclei. The subcellular distribution behavior of the NC compounds in the three cells was measured using a confocal laser scanning microscope. As shown in Figures S12–S16, strong green and blue fluorescence was observed in the nuclei, clearly indicating that M-3 was mainly located in the nucleus. While the presence of the PEG chain in the structure provided electrons for the conjugated host, the NC-5-3O emission wavelength was slightly red-shifted compared with the other compounds, shifting from a green to a yellow fluorescence. In short, all results demonstrated the efficient cellular uptake of the NC compounds.

## 3. Experimental Section

### 3.1. Materials and Instruments

All reagents used in the experimental section were spectroscopic grade and were purchased from Leyan Co., Ltd. (Shanghai, China), Macklin Biochemical Co., Ltd. (Shanghai, China), and Aladdin Co., Ltd. (Shanghai, China). Also, Bovine serum albumin (BSA) and PEG400 were obtained from Shanghai Macklin Biochemical Co., Ltd. Furthermore, the confocal images mentioned were captured by a LEICA TCS SP8 Confocal Microscope System (Wetzlar, Germany). UV–visible absorption spectra and fluorescence emission spectra were determined at room temperature at concentrations of around 50 μM with a SHIMADZU UV-3600 Plus Spectrophotometer (Shimadzu, Kyoto, Japan) and an Edinburgh Instruments FLS980 fluorescence spectrometer (Edinburgh, Livingston, UK) with slit widths routinely set to 3 nm. Fluorescence lifetime was detected on an A1 fluorescence lifetime microscope system (Tokyo, Japan). NMR spectra were recorded on a Bruker DRX-400 MHz spectrometer (Ettlingen, Germany). Chemical shifts were reported in ppm, and coupling constants (J) were reported in Hz. High-performance liquid chromatography (HPLC) analysis was performed at room temperature using Nexera UHPLC LC-30A (Shimadzu, Japan).

### 3.2. Synthetic Procedures for NC Compounds

**General procedures for the preparation of compound NC-2.** Tetrabutylammonium acetate (570 mmol; 1.0 eq.) was dissolved in 15 mL THF at r.t. After the mixture was thoroughly stirred for 5 min, CH_3_I (2 mL; 15.0 eq.) was slowly added by syringe, and KOH-saturated aqueous solution (1.5 mL, 2.0 equiv.) was generally added to the above mixture. Since the whole system released heat, the mixture was placed in a cold-water bath and cooled to room temperature, and compound NC-1 (2 g; 11.36 mmol; 6.0 eq.) was added. The whole mixture reacted at 25 °C for 8 h. The mixture was extracted with ethyl acetate (3 × 85 mL). The extracted organic layer was then dried over Na_2_SO_4_, filtrated, and concentrated to dryness. The crude product was purified by column chromatography on silica gel (petroleum ether:ethyl acetate = 35:1 to 20:1) to obtain compound NC-2 as a colorless transparent oily liquid (1.18 g, 52%).

**General procedures for the preparation of compound NC-3.** Compound NC-2 (1.18 g; 5.8 mmol; 1.0 eq.) was dissolved in DMSO (8 mL). After the mixture was stirred for 3 min, IBX (1.3 g; 4.62 mmol; 0.8 eq.) was added to the solution. Then, the mixture was heated under 80 °C for 10 h. At the end of the reaction, the mixture was cooled to r.t. and was extracted with ethyl acetate (3 × 90 mL). The extracted organic layer was washed with saturated NaCl solution (65 mL) and then dried over Na_2_SO_4_, filtrated, and concentrated to dryness. The product was purified by column chromatography on silica gel (PE:EA = 15:1) to collect compound NC-3 as a white solid (993 mg, 85%).

**General procedures for the preparation of compound NC-3-Br.** Compound NC-3 (263.4 mg; 1.0 eq.) was dissolved in 13 mL MeCN and NBS (1.4 g; 4.5 mmol; 3.0 eq.). The mixture reacted at room temperature for 4 h. Then, the reaction mixture was purified by column chromatography on silica gel (PE:EA = 8:1) to collect compound NC-3-Br as a mild yellow solid (116 mg, 28%).

**General procedures for the preparation of compound NC-3-Ph.** Compound NC-3 (300 mg; 1.5 mmol; 1.0 eq.) was dissolved in 15 mL DMF, and phenylboronic acid (198 mg; 1.1 eq.), 5-Nitro-1, 10-phenanthroline (160 mg; 1.1 eq.), and Pd(OAc)_2_ (166 mg; 0.5 eq.) were added to the solution. After all the medicines were added, the gas in the system was replaced with O_2_, and the reaction was heated and stirred at 80 °C for 9 h. At the end of the reaction, the mixture was cooled to r.t. and was extracted with ethyl acetate (3 × 40 mL). The extracted organic layer was washed with saturated NaCl solution (30 mL) and then dried over Na_2_SO_4_, filtrated, and concentrated to dryness. The crude product was purified by column chromatography on silica gel (PE:EA = 10:1) to collect compound NC-3-Ph as a mild yellow solid (131.6 mg, 32%).

**General procedures for the preparation of compound NC-4.** Compound NC-3 (1 g, 4.9 mmol; 1.0 eq.) was dissolved in toluene (20 mL). AcOH (2.5 mL; 0.5 mmol; 0.1 eq.) and NH_4_OAc (185 mg; 3.96 mmol; 0.8 eq.) were added to the solution. Then, the mixture was stirred for 30 min, and malononitrile (1.3 g; 19.8 mmol; 4.0 eq.) was added. The reaction mixture was heated under reflux (90 °C) for 20 h. At the end of the reaction, the mixture was cooled to r.t. and vacuum-concentrated, and the crude product was purified by column chromatography on silica gel (PE:EA = 15:1 to 10:1) to collect compound NC-4 as a yellow solid (592 mg, 48%).

**General procedures for the preparation of compounds NC-4-F, NC-4-Br, and NC-4-Ph.** The procedures were the same for the preparation of NC-4. NC-4-F, NC-4-Br, and NC-4-Ph, which were collected as yellow solids, 48 mg, 38%; 47 mg, 41%; and 38.7 mg, 33%, respectively.

**General procedures for the preparation of compound NC-5-IBX.** In a mixture of ice and water at 0 °C, NaH (1 g; 40 mmol; 10.0 eq.) was dissolved in 12 mL of N, N-dimethylformamide (DMF). Under a nitrogen atmosphere, NaSH (2.5 g; 40 mmol; 10.0 eq.) was added dropwise to the reaction solvent, and the system was thoroughly mixed and stirred for 5 min in an ice bath. NC-4 (1 g; 4 mmol; 1.0 eq.) was then added to the reaction mixture and stirred until it reached room temperature. The ice bath was removed, and the oil bath was substituted at 120 °C for 6 h. After a complete reaction, the mixture was cooled to room temperature, and the solvent was replaced with a water bath at ambient temperature. Slow dropwise addition of 8 mL of 4 M HCl to the reaction solution was carried out while the mixture was stirred for approximately 20 min until the pH reached ≤7. After complete neutralization, the mixture was extracted three times with ethyl acetate (3 × 85 mL). The organic layer was dried with anhydrous Na_2_SO_4_, filtered, and then concentrated via rotary evaporation to obtain the crude product. The crude product (300 mg; 1.3 mmol; 1.0 eq.) was dissolved in DMSO (10 mL) and stirred thoroughly. IBX (CAS: 61717-82-6; 356 mg; 1.3 mmol; 1.0 eq.) was added in portions to the solution. The mixture was reacted at 80 °C in an oil bath for 8 h. After the completion of the reaction, the mixture was cooled to room temperature and extracted three times with ethyl acetate (3 × 90 mL). The organic layer was washed with saturated NaCl solution (45 mL) and dried with anhydrous Na_2_SO_4_. After filtration and subsequent vacuum distillation, the crude product was obtained. The crude product was purified by silica gel column chromatography (petroleum ether:ethyl acetate = 12:1) to afford compound NC-5-IBX as an orange solid (134 mg, 45%).

**General procedures for the preparation of compound NC-5-3O.** NC-5-IBX (80 mg; 0.3 mmol; 1.0 eq.) was dissolved in DMF, followed by K_2_CO_3_ (119 mg; 0.9 mmol; 2.5 eq.) and 2-(2-(2-methoxyethoxy) ethoxy) ethyl 4-methylbenzenesulfonate (120 mmol; 0.4 mmol; 1.2 eq.). The reaction flask was placed in an oil bath and heated under 100 °C for 10 h. After extraction, the mixture was purified by silica gel column chromatography (petroleum ether:ethyl acetate = 5:1), and the crude product was collected as a pale yellow solid. To further purify the compound, an HPLC (high-performance liquid semi-automatic separation column) was used to obtain pure NC-5-3O (12 mg, 21%).

### 3.3. Optical Properties Measurements

UV–visible absorption spectra and fluorescence emission spectra were determined at room temperature at concentrations of around 50 μM with a SHIMADZU UV-3600 Plus Spectrophotometer (Kyoto, Japan) and an Edinburgh Instruments FLS980 fluorescence spectrometer (Livingston, UK) with slit widths routinely set to 3 nm.

### 3.4. Fluorescence Lifetime Measurements

The fluorescence lifetime measurements in the solvents were conducted using an F-7000 fluorescence spectrometer (Hitachi, Japan), which was equipped with a pulsed diode laser. The mean-weighted fluorescence lifetime was determined by considering both lifetime components (τ_i_) and their corresponding amplitudes (α). The calculation was performed according to the provided equation.

τ = (τ_1_^2^ α_1_ + τ_2_^2^ α_2_)/(τ_1_α_1_ + τ_2_α_2_) (1)

### 3.5. Molecular Orbitals Calculations

All calculations were performed using the Gaussian 16 software. B3LYP functional, 6-31G (d, p) basis set, and the IEFPCM solvent model were used to optimize the molecular structure and calculate properties in different solvents. TD-B3LYP was used to calculate the excitation energy and optimize the structure of the first excited state.

### 3.6. Cell Culture

Healthy human gastric mucosal cell line (GES-1), human osteosarcoma cells (SJSA-1), human colorectal adenocarcinoma cells (HCT-8), and colon cancer cell line MC-38 were purchased from the cell bank of the Chinese Academy of Sciences (Shanghai, China). All cells were cultured in RPMI-1640 media. All cell lines were provided by the lab of Prof. Xiongwen Zhang and Dr. Suzheng Dong.

### 3.7. In Vitro Cellular Uptake

In order to investigate the cellular uptake behavior of these compounds, cells were seeded in 20 mm glass-bottomed culture dishes at appropriate densities and incubated for 24 h. Subsequently, varying concentrations of these compounds were added equally to the aforementioned cells, followed by incubation for different durations. After washing the cells with PBS, they were fixed with 4% paraformaldehyde and visualized using a Leica TCS SP8 confocal fluorescence microscope from Germany.

## 4. Conclusions

In the fluorescence tests, the fluorescence emission of the NC compounds failed to exceed 700 nm, which was also shown by the confocal imaging results. These compounds are favorable green fluorescent dyes, and no quenching was observed when the NC compounds were tested in an aqueous environment. However, the fluorescence emission intensity in water was higher than in organic solvents. During the confocal imaging of cancer cells, the compounds efficiently stained the cell nuclei and emitted green fluorescence. Considering that the synthesized compounds also have good fluorescence and luminescence properties in PBS and ultrapure water, we reasonably determined that the NC compounds could interact with pathological living cells, such as those found in colon cancer and osteosarcoma. Among all the compounds, NC-4-Br had a long fluorescence lifetime, high chemical stability, a strong fluorescence intensity, and a considerable excitation–emission gap. For this reason, NC-4-Br showed the best potential as a green fluorescent small molecule among the NC compounds that can effectively stain living cells.

## Figures and Tables

**Table 1:** NC-4-Br spectral data.

Solvent	$\lambda_{b}^{a}$ (nm)	$\lambda_{c}^{a}$ (nm)	Δλ (nm)	Q_Ein_	AF
EA	427	522	95	0.1097	0.0412
DCM	432	525	93	0.1105	0.0570
EtOH	426	537	111	0.1251	0.0372
MeOH	427	538	111	0.1103	0.0892
MeCN	425	534	109	0.1130	0.0372
DMSO	438	544	106	0.1131	0.0424
PBS	440	547	107	0.8512	0.0318
H_2_O	437	551	114	0.1078	0.0604

^a^ maximum; ^b^ absorption; ^c^ emission.

**Table 2:** Fluorescence decay of compounds in DMSO and H_2_O.

DMSO	Lifetime (ns)	Chisqr	H_2_O	Lifetime (ns)	Chisqr
NC-4	0.4865	1.1646	NC-4	0.7109	1.2095
NC-4-F	1.1789	1.4828	NC-4-F	0.8214	1.2838
NC-4-Br	4.6383	1.0791	NC-4-Br	5.9386	1.0211
NC-4-Ph	3.5154	1.0572	NC-4-Ph	2.9349	1.4302
NC-5-IBX	4.1780	1.1997	NC-5-IBX	2.0648	1.1008
NC-5-3O	4.0990	1.1743	NC-5-3O	0.9555	1.0602

## Data Availability

The data that support this study will be shared upon reasonable request to the corresponding authors.

## Supplementary Materials

The following supporting information can be downloaded at https://www.mdpi.com/article/10.3390/molecules29143396/s1. Synthesis of compounds; Spectral data of the compound; Fluorescence emission spectra of compounds in different solvents; Cytotoxicity of the compound to GES-1 cells; In vitro cellular uptake of the compound in three cancer cell lines; ^1^H-NMR, ^13^C-NMR and purity of compounds.
