# Supplementary material for: Design, Synthesis, and Evaluation of Small Fluorescent Molecules with a 1,1-Dimethylnaphthalen-2-(1H)-One Core
Source: Molecules. 2024 Jul 19;29(14):3396. doi: 10.3390/molecules29143396 (PMC11280428; doi:10.3390/molecules29143396)
Supplement: Supplementary file 1 [file molecules-29-03396-s001.zip › molecules-3046824-supplementary.pdf]

Synthesis of target compounds.

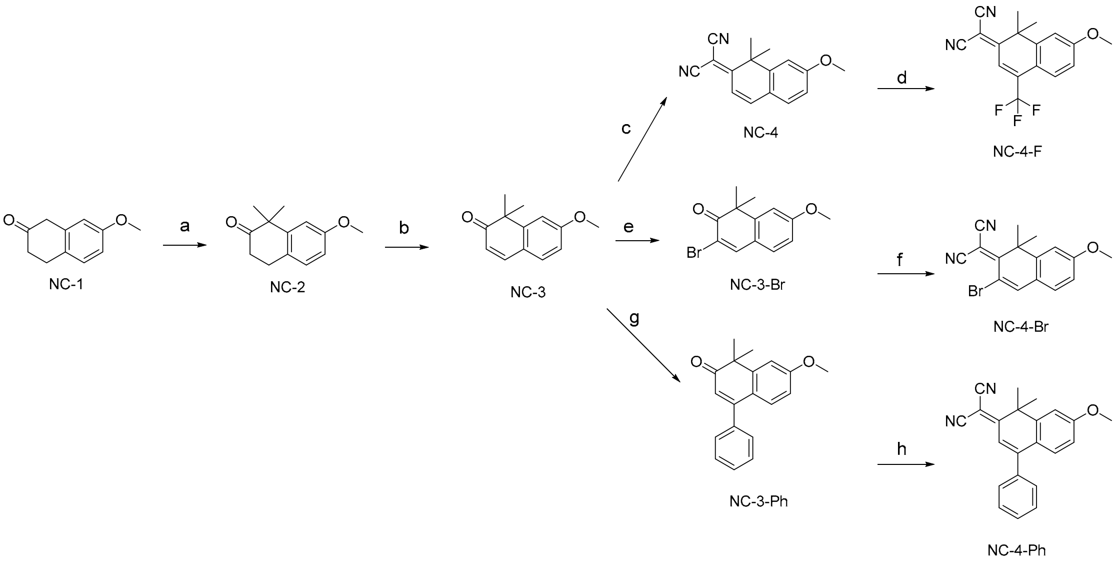

Scheme S1. Reagents and conditions: (a) **NC-1** (7-methoxy-3,4-dihydronaphthalen-2(1H)-one), CH_3I, tetrabutylammonium acetate, KOH (aq), THF, r.t., 8 h; (b) IBX, DMSO, 80 °C, 10 h; (c) malononitrile, NH_4OAc, NH_4OAc, toluene, 90 °C, 20h; (d) 1-trifluoroMethyl-1,2-benziodoxol-3(1H)-one, CuI, 80 °C, 8 h; (e) NBS, N_2, MeCN, r.t., 4 h; (f) malononitrile, NH_4OAc, toluene, 90 °C, 18 h; (e) phenyl boronic acid, 5-nitro-1,10-phenanthroline, $Pd(OAc)_2$, O_2, DMF, 80 °C, 9 h; (h) malononitrile, NH_4OAc, NH_4OAc, toluene, 90 °C, 18 h.

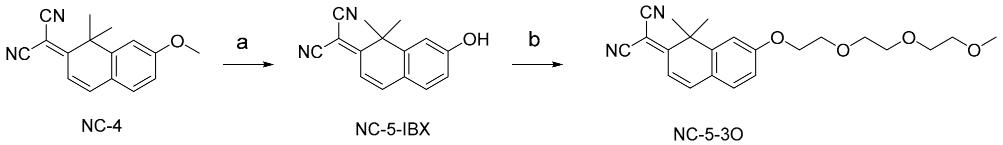

Scheme S2. Reagents and conditions: (a) IBX, DMSO, 80 °C; (b) 2-(2-(2-methoxyethoxy)ethoxy)ethyl 4-methylbenzenesulfonate, K_2CO_3, DMF, 100 °C , 10 h.

Table S1. NC-4 spectrum data.

**Solvent**	**λ_b^a(nm)**	**λ_c^a(nm)**	**Δλ(nm)**	**Q_{Ein}**	**AF**
EA	422	527	105	0.0316	0.1633
DCM	425	528	103	0.0681	0.1313
EtOH	426	530	104	0.0583	0.1509
MeOH	424	530	106	0.0549	0.1399
MeCN	423	527	104	0.0370	0.1509
DMSO	434	536	102	0.0386	0.1339
PBS	426	545	119	0.0217	0.0923

H_2O	425	545	120	0.0426	0.1079

[a] maximum
[b] absorption
[c] emission

Table S2. NC-4-F spectrum data.

**Solvent**	**λ_b^a(nm)**	**λ_c^a(nm)**	**Δλ(nm)**	**Q_{Ein}**	**AF**
EA	424	539	115	0.0318	0.0651
DCM	429	539	110	0.0419	0.1912
EtOH	429	552	123	0.0512	0.1890
MeOH	424	553	129	0.0301	0.1689
MeCN	425	557	132	0.0278	0.1890
DMSO	431	573	142	0.0488	0.0519
PBS	430	536	106	0.271	0.0381
H_2O	428	531	103	0.0339	0.0589

[a] maximum
[b] absorption
[c] emission

Table S3. NC-4-Ph spectrum data.

**Solvent**	**λ_b^a(nm)**	**λ_c^a(nm)**	**Δλ(nm)**	**Q_{Ein}**	**AF**
EA	433	540	107	0.0613	0.1337
DCM	437	538	101	0.0412	0.1139
EtOH	438	549	111	0.0383	0.1267
MeOH	431	550	119	0.0491	0.2036
MeCN	434	542	108	0.0523	0.1267
DMSO	442	534	92	0.0543	0.1172
PBS	434	574	140	0.04182	0.0621
H_2O	436	568	132	0.0543	0.1172

[a] maximum
[b] absorption
[c] emission

Table S4. NC-5-IBX spectrum data.

**Solvent**	**λ_b^a(nm)**	**λ_c^a(nm)**	**Δλ(nm)**	**Q_{Ein}**	**AF**
EA	437	510	73	0.0608	0.0935
DCM	437	515	78	0.0409	0.1395
EtOH	439	521	82	0.0431	0.1405
MeOH	432	520	88	0.0477	0.1381
MeCN	434	518	84	0.0289	0.1405
DMSO	432	526	94	0.0533	0.1015
PBS	434	618	184	0.0428	0.1291
H_2O	435	614	179	0.0432	0.1150

[a] maximum
[b] absorption

[c] emission

Table S5. NC-5-3O spectrum data.

Solvent	λ_b^a(nm)	λ_c^a(nm)	$\Delta\lambda$(nm)	Q_{Ein}	AF
EA	436	513	77	0.0383	0.0984
DCM	437	516	79	0.0513	0.1330
EtOH	439	514	75	0.0610	0.0894
MeOH	433	523	90	0.0370	0.0916
MeCN	436	519	83	0.0293	0.0894
DMSO	436	519	83	0.0233	0.0742
PBS	435	619	184	0.0281	0.1218
H_2O	437	616	179	0.0389	0.0683

[a] maximum
[b] absorption
[c] emission

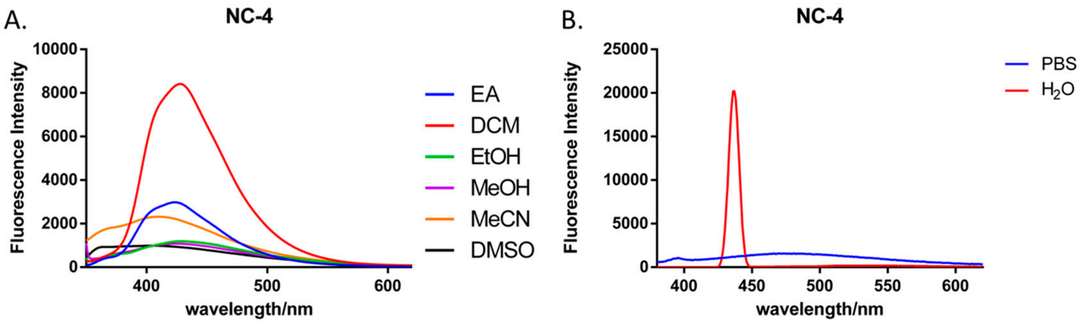

Figure S1. Fluorescence emission spectrum of NC-4 in different solvents.

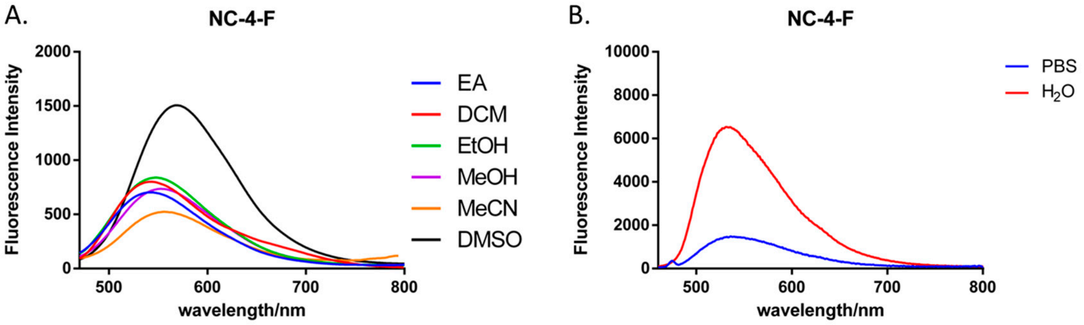

Figure S2. Fluorescence emission spectrum of NC-4-F in different solvents.

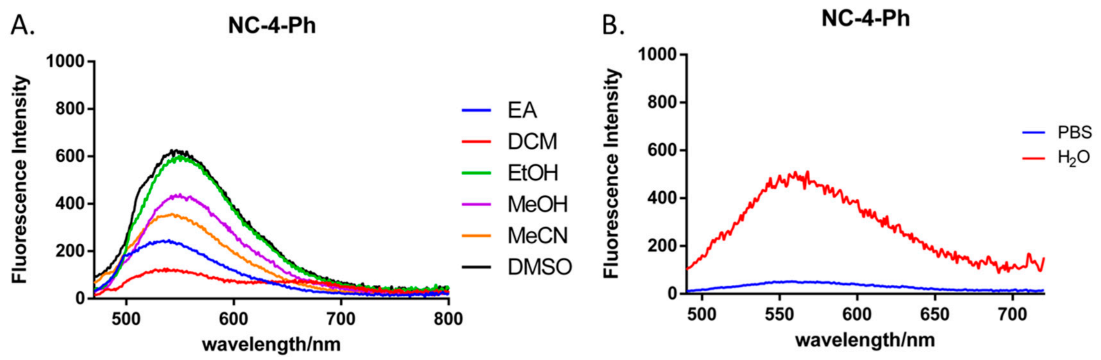

Figure S3. Fluorescence emission spectrum of NC-4-Ph in different solvents.

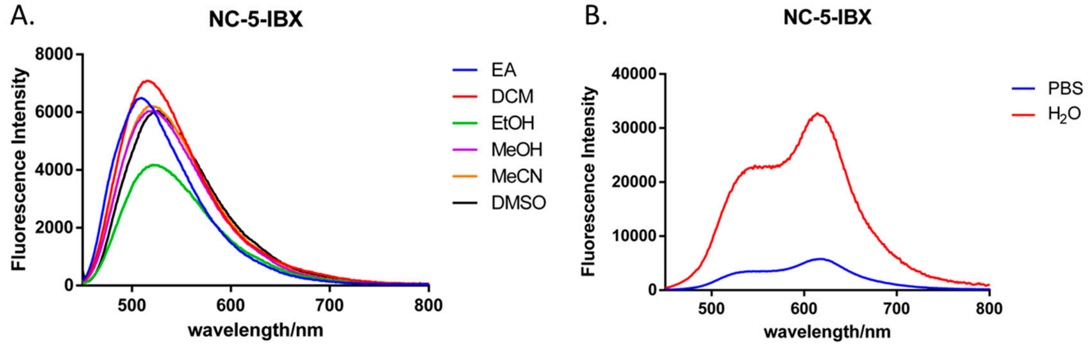

Figure S4. Fluorescence emission spectrum of NC-5-IBX in different solvents.

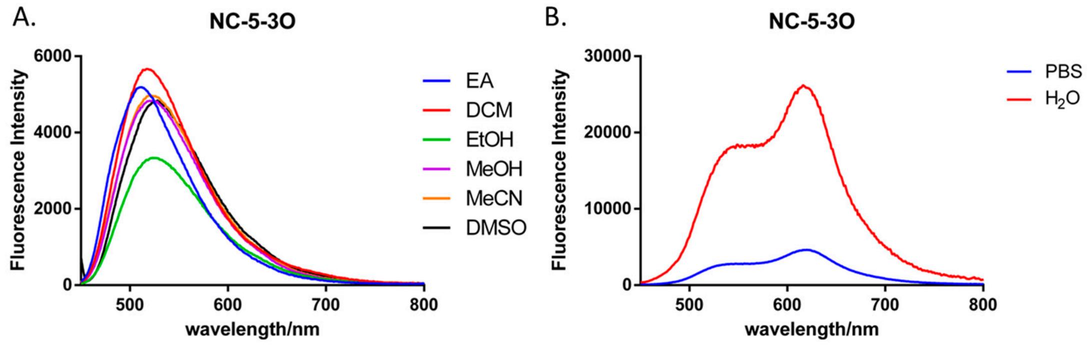

Figure S5. Fluorescence emission spectrum of NC-5-3O in different solvents.

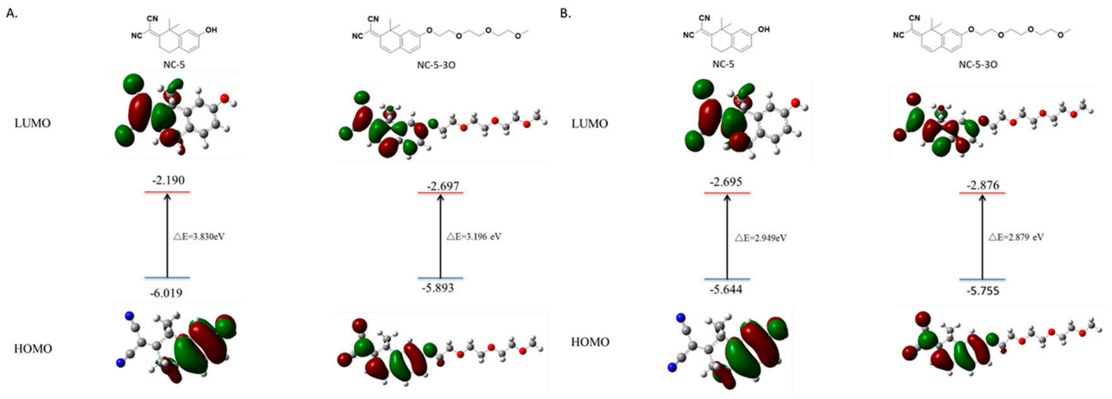

Figure S6. Frontier orbital distributions, energy levels calculated by Gaussian 16 in the ground state and first excited state. A) Ground state; B) First excited state.

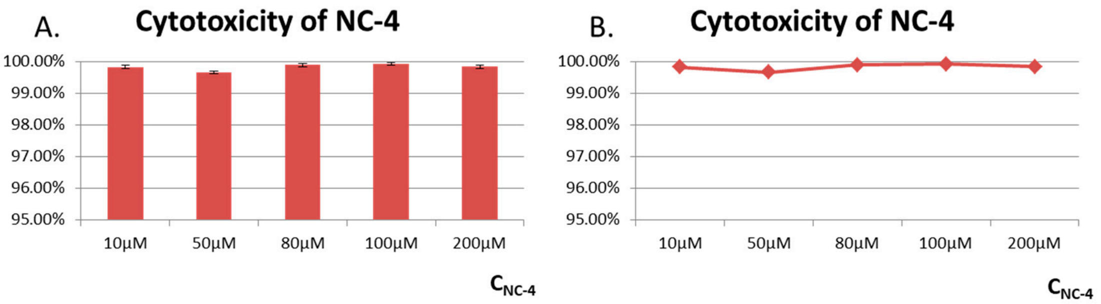

Figure S7. Cytotoxicity of NC-4 in GES-1 cells.

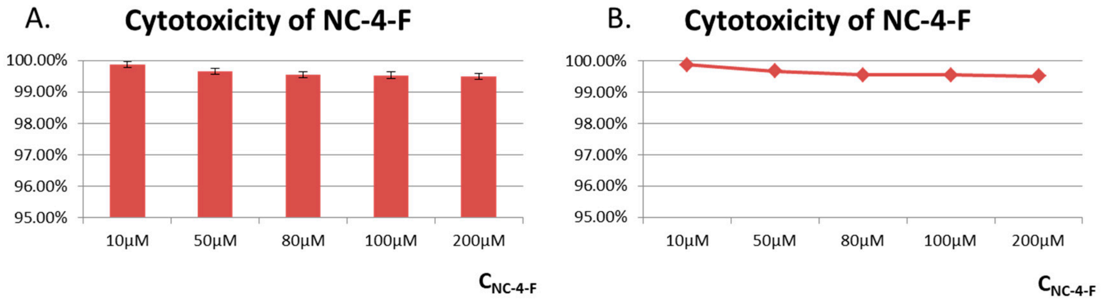

Figure S8. Cytotoxicity of NC-4-F in GES-1 cells.

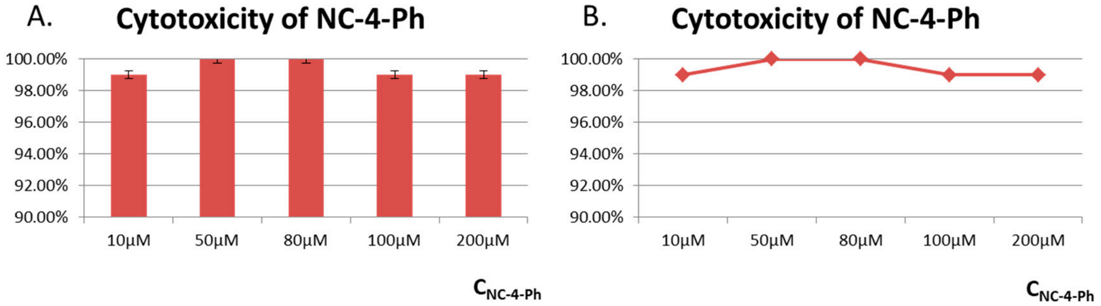

Figure S9. Cytotoxicity of NC-4-Ph in GES-1 cells.

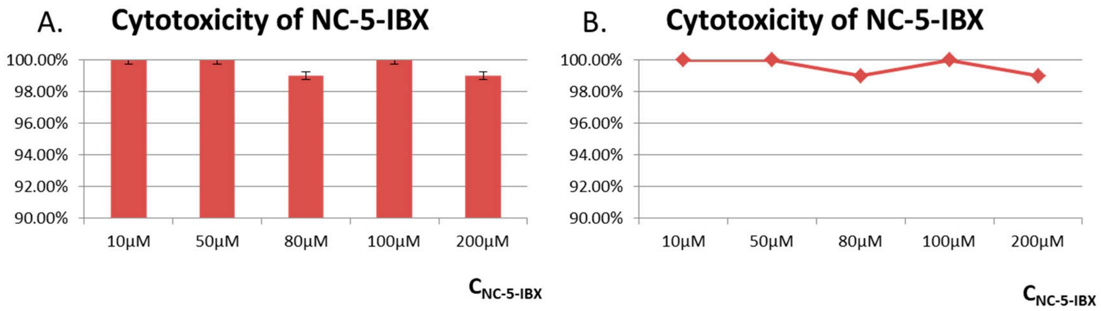

Figure S10. Cytotoxicity of NC-5-IBX in GES-1 cells.

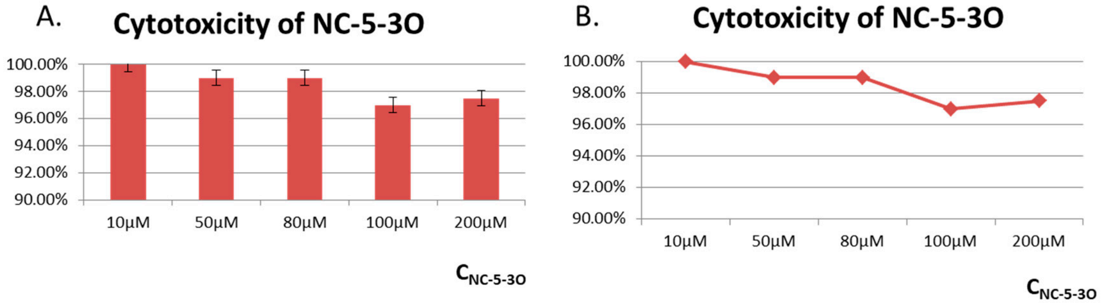

Figure S11. Cytotoxicity of NC-5-3O in GES-1 cells.

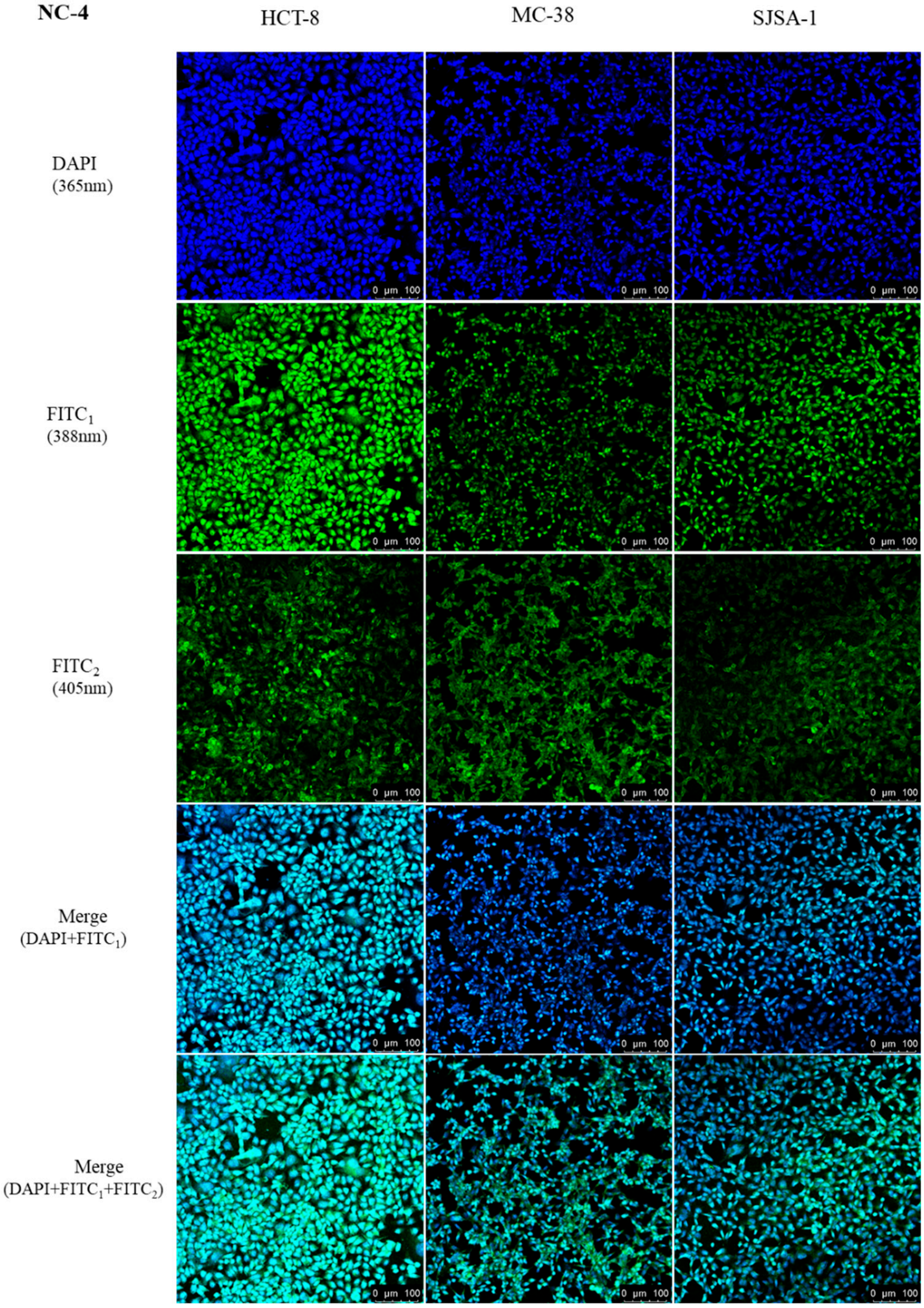

Figure S12. In vitro cellular uptake of NC-4 in three cancer cell lines.

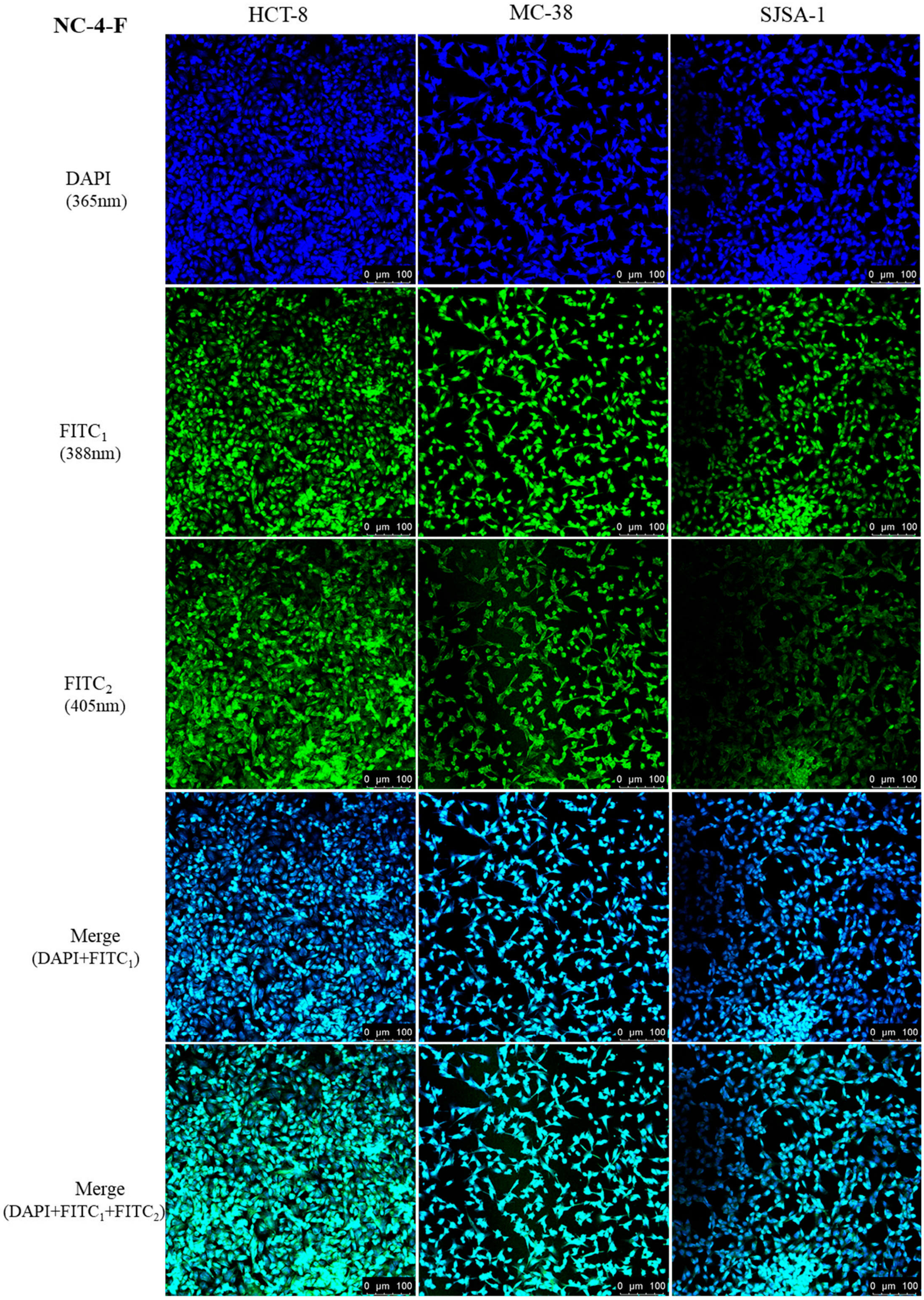

Figure S13. In vitro cellular uptake of NC-4-F in three cancer cell lines.

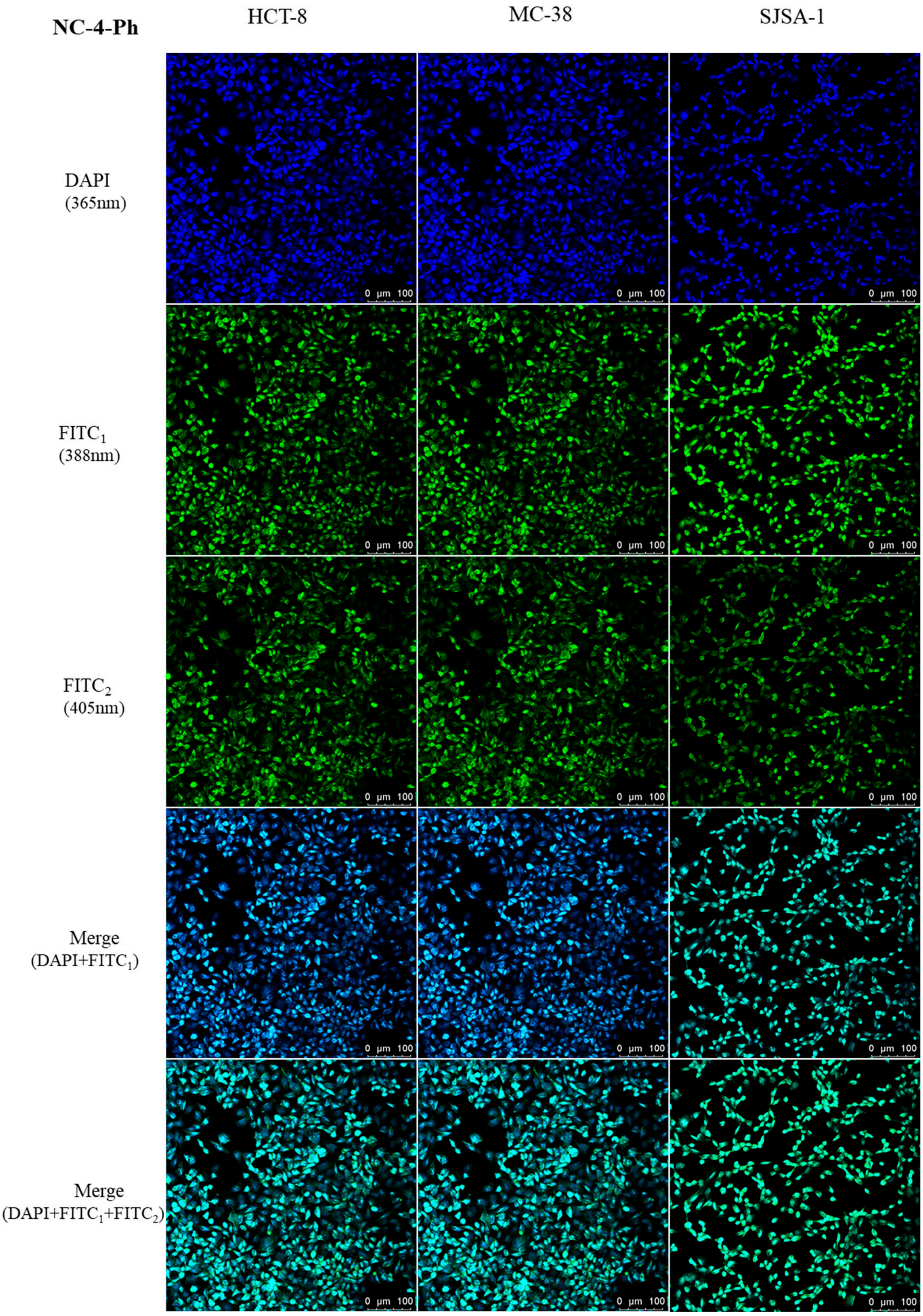

Figure S14. In vitro cellular uptake of NC-4-Ph in three cancer cell lines.

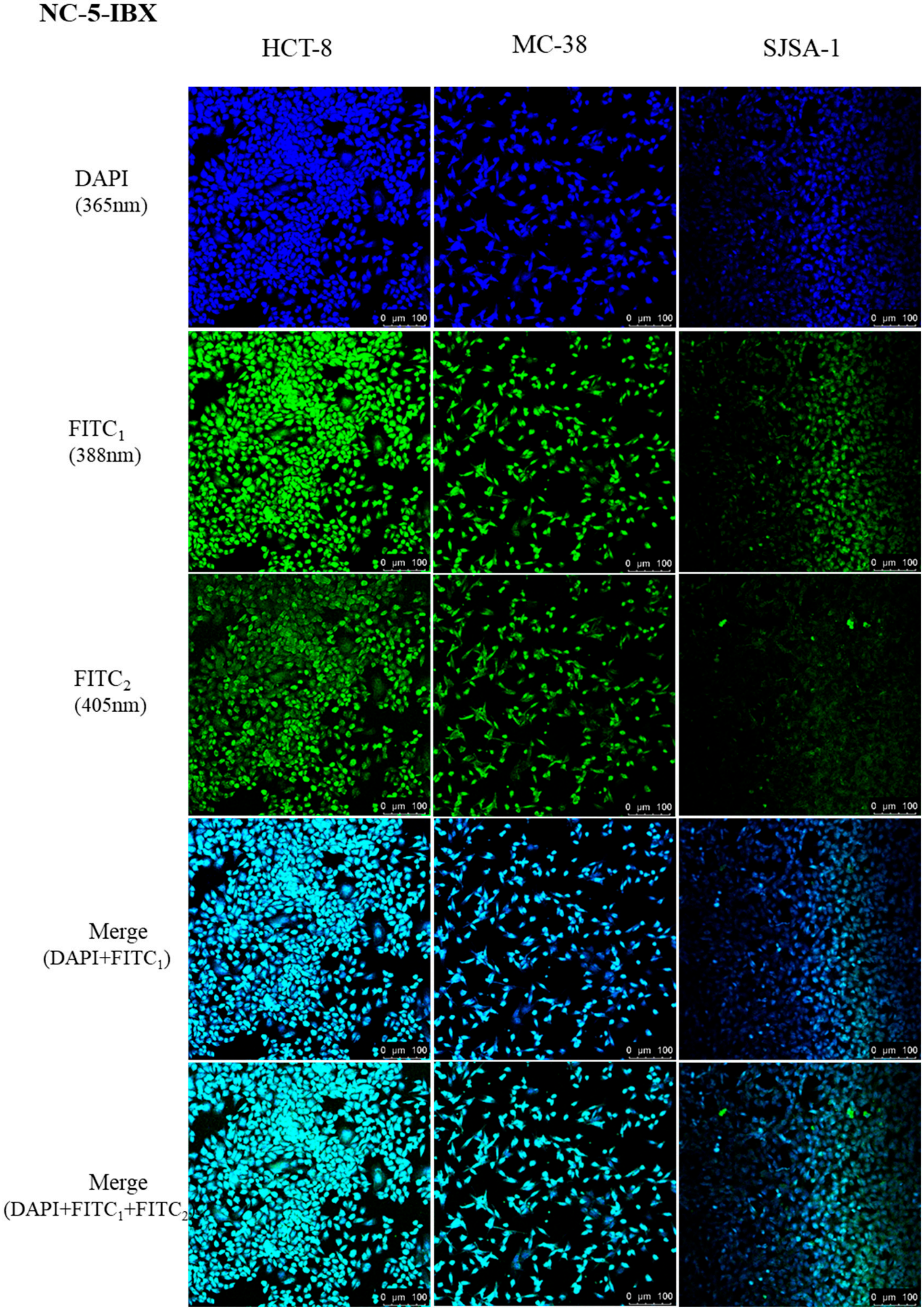

Figure S15. In vitro cellular uptake of NC-5 in three cancer cell lines.

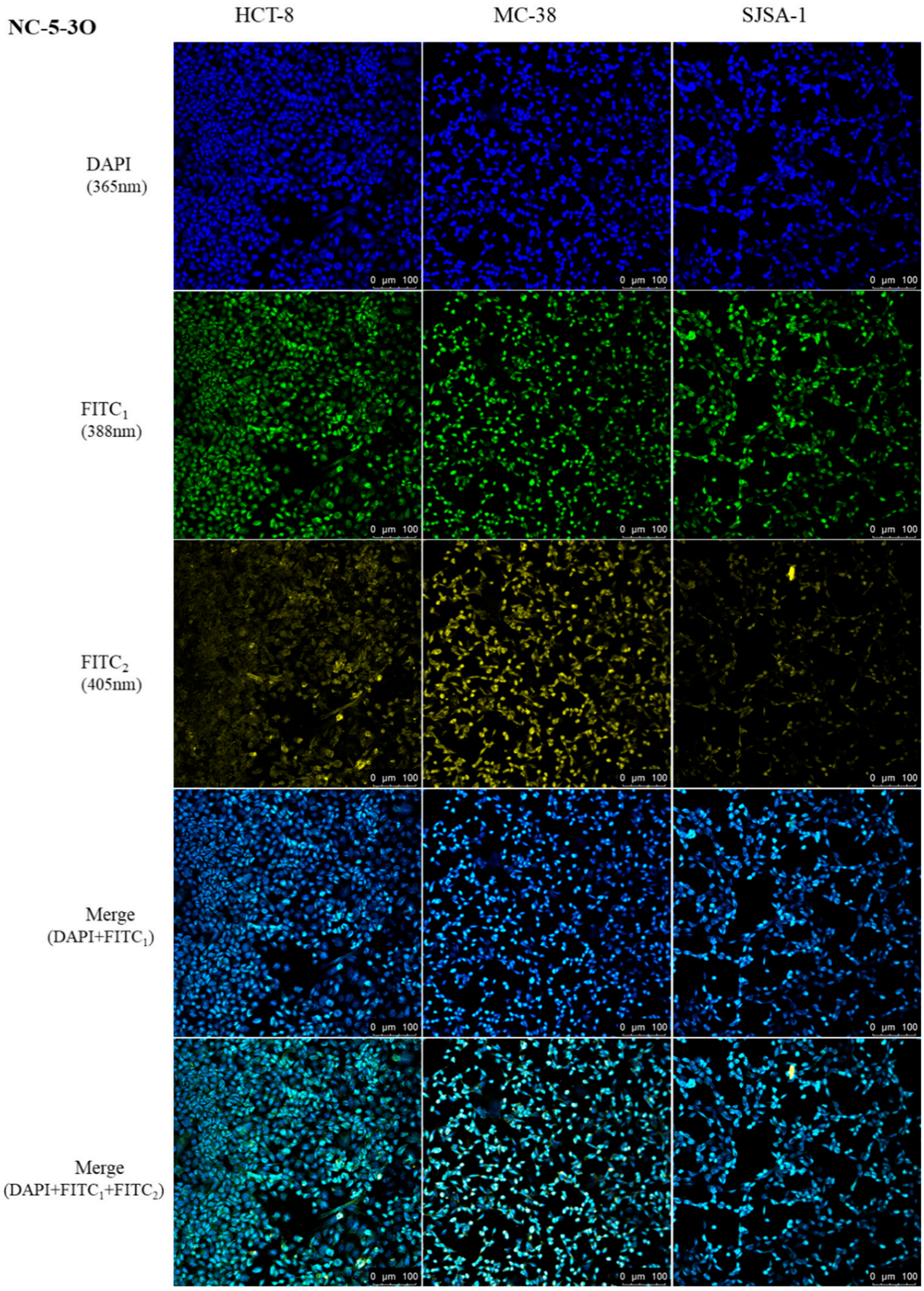

Figure S16. *In vitro* cellular uptake of NC-5-3O in three cancer cell lines.

NMR spectra

NC-2

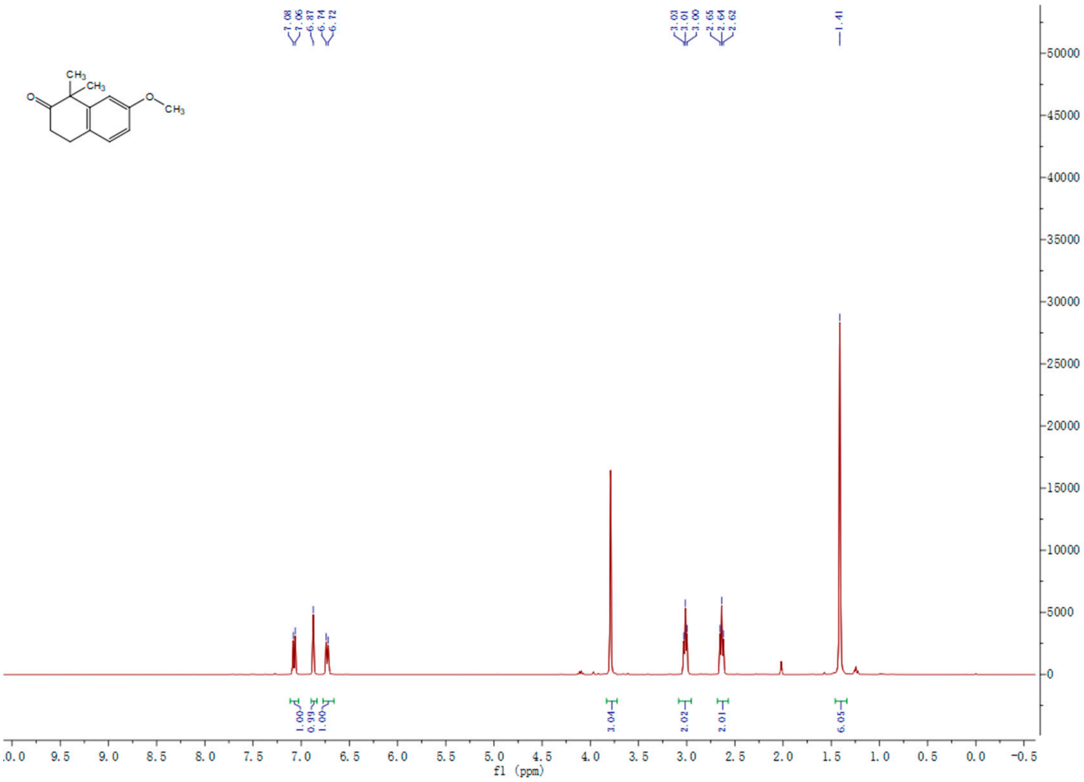

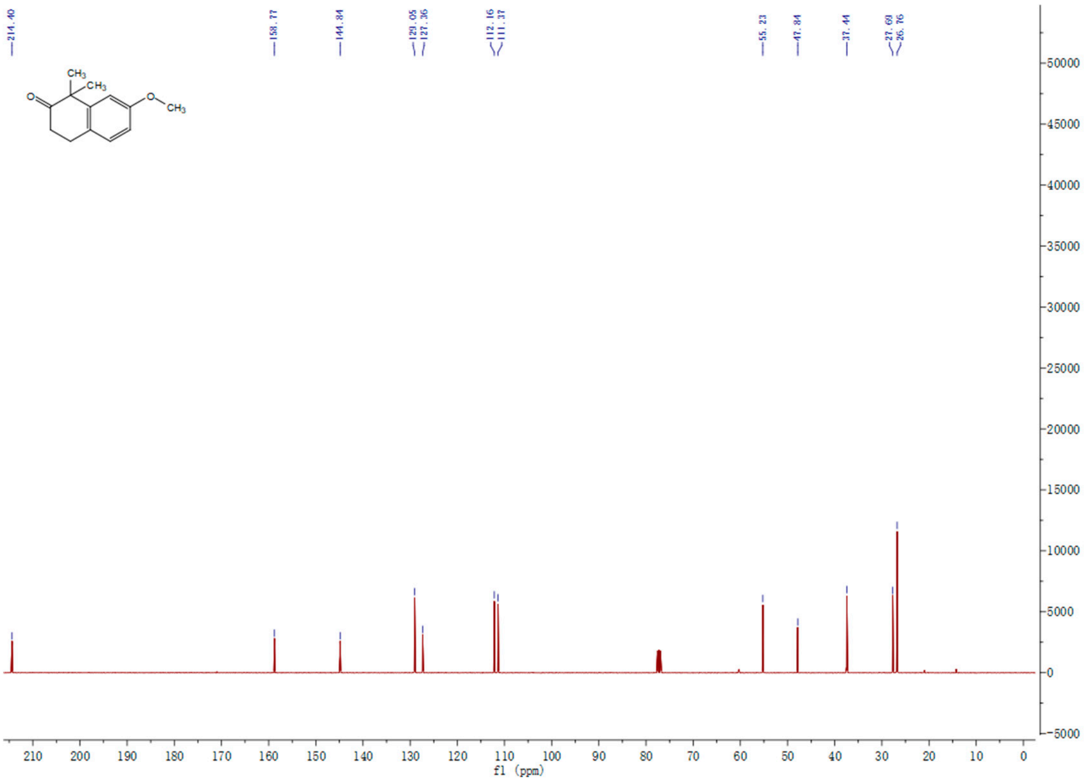

NC-3

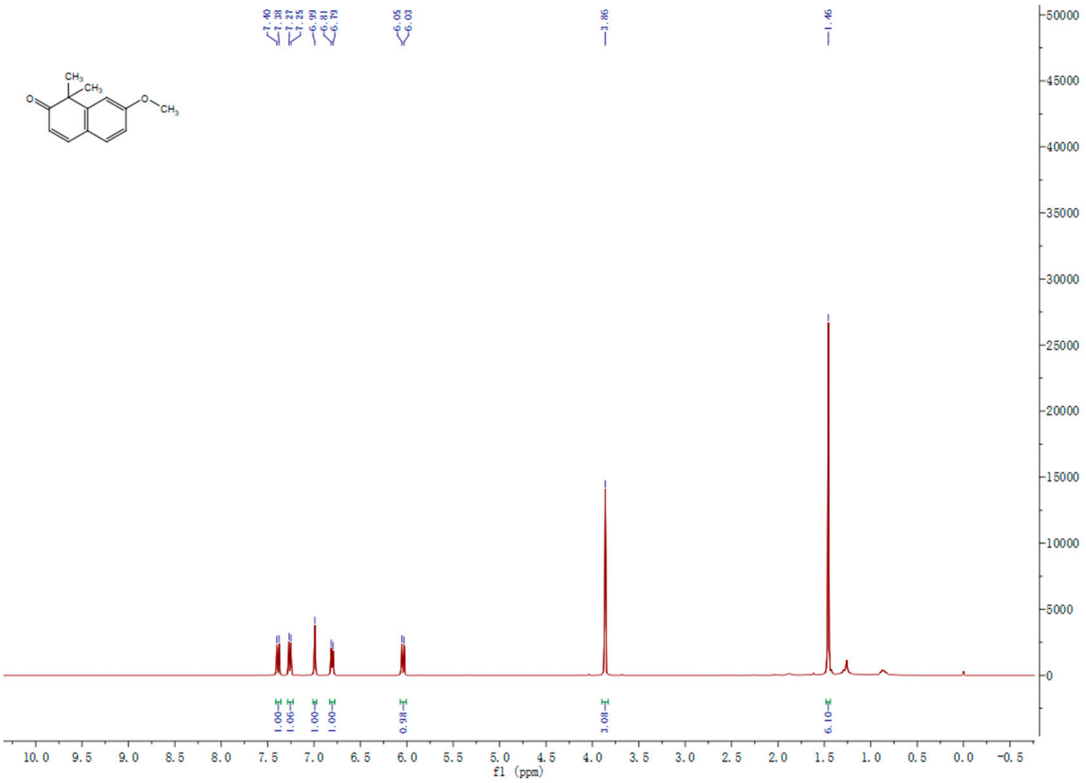

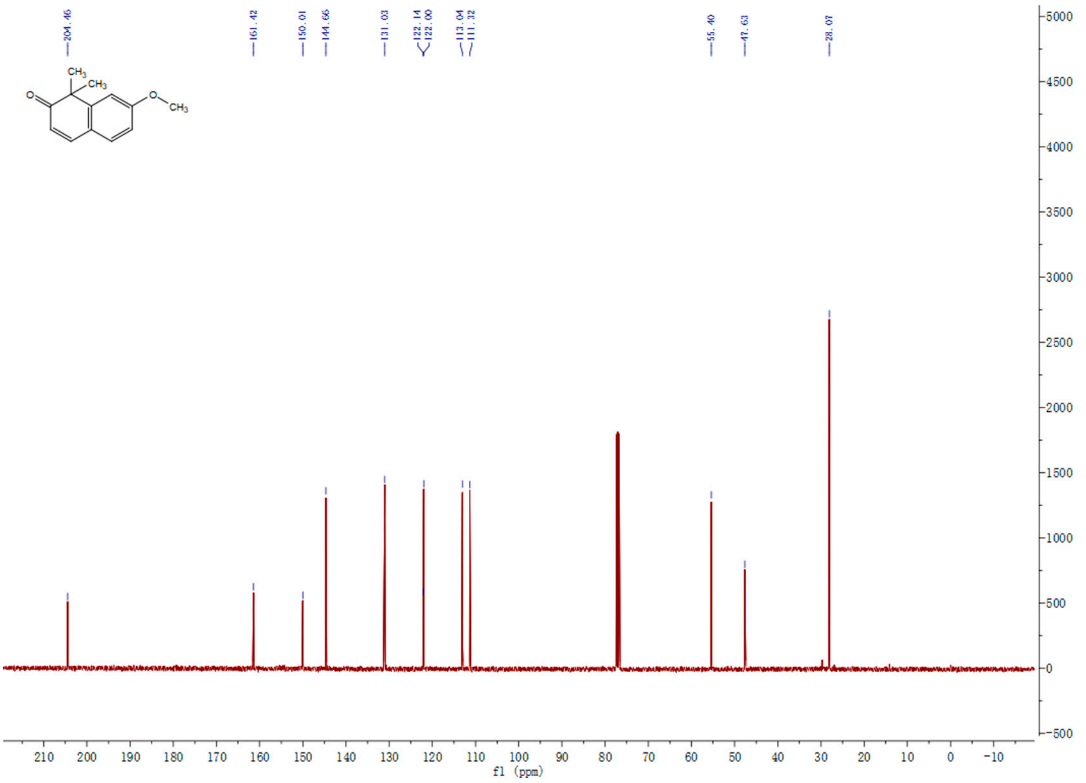

NC-4

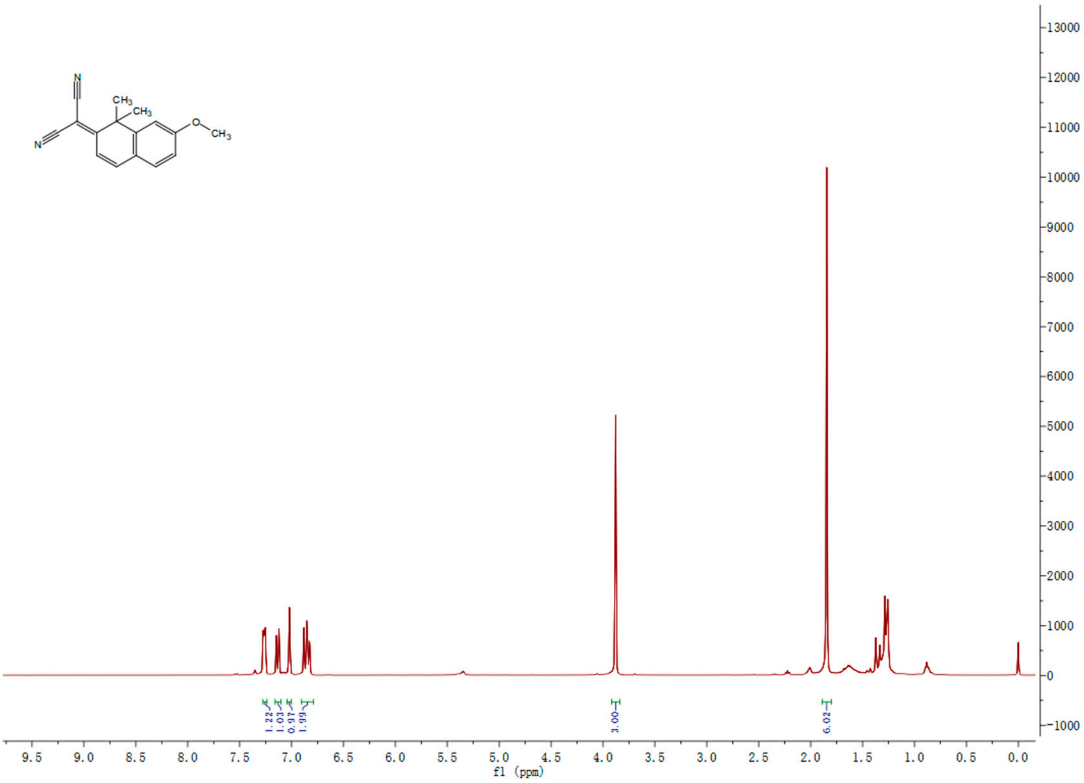

N
CH3
CH3
O
CH3
N
1.22
1.03
0.97
1.99
3.00
6.02
f1 (ppm)

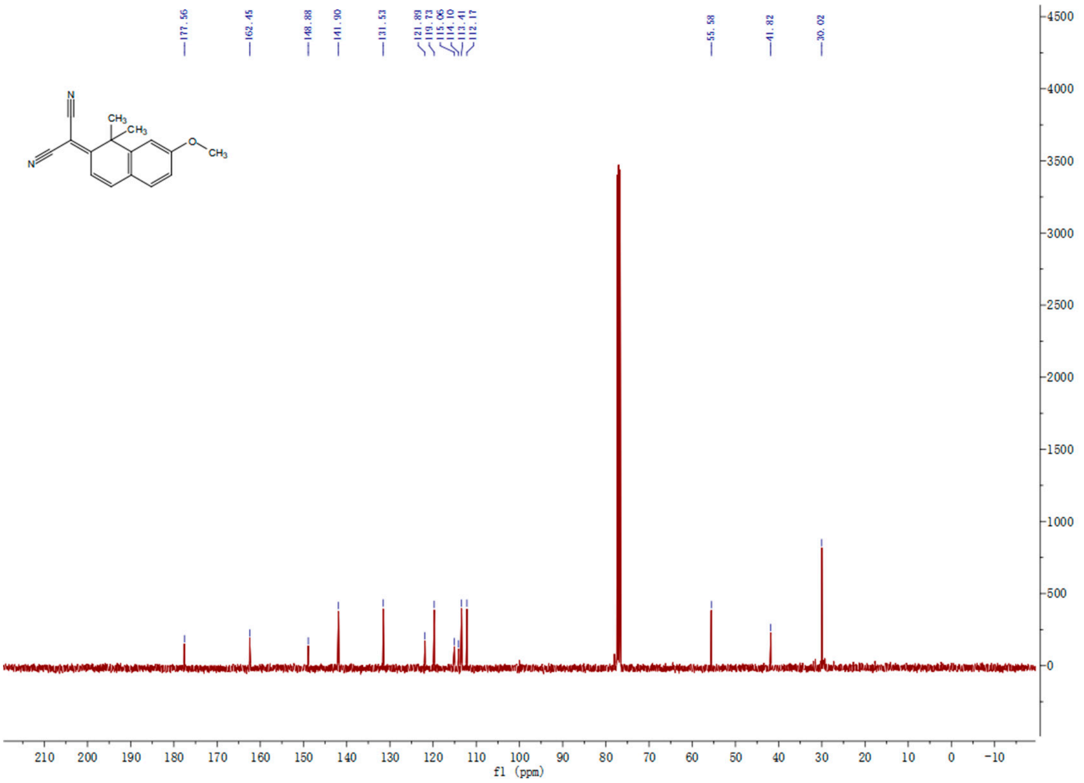

177.56
162.45
148.88
141.90
131.53
121.89
119.73
115.06
114.10
113.41
112.17
55.58
41.82
30.02
N
CH3
CH3
O
CH3
N
f1 (ppm)

NC-4-F

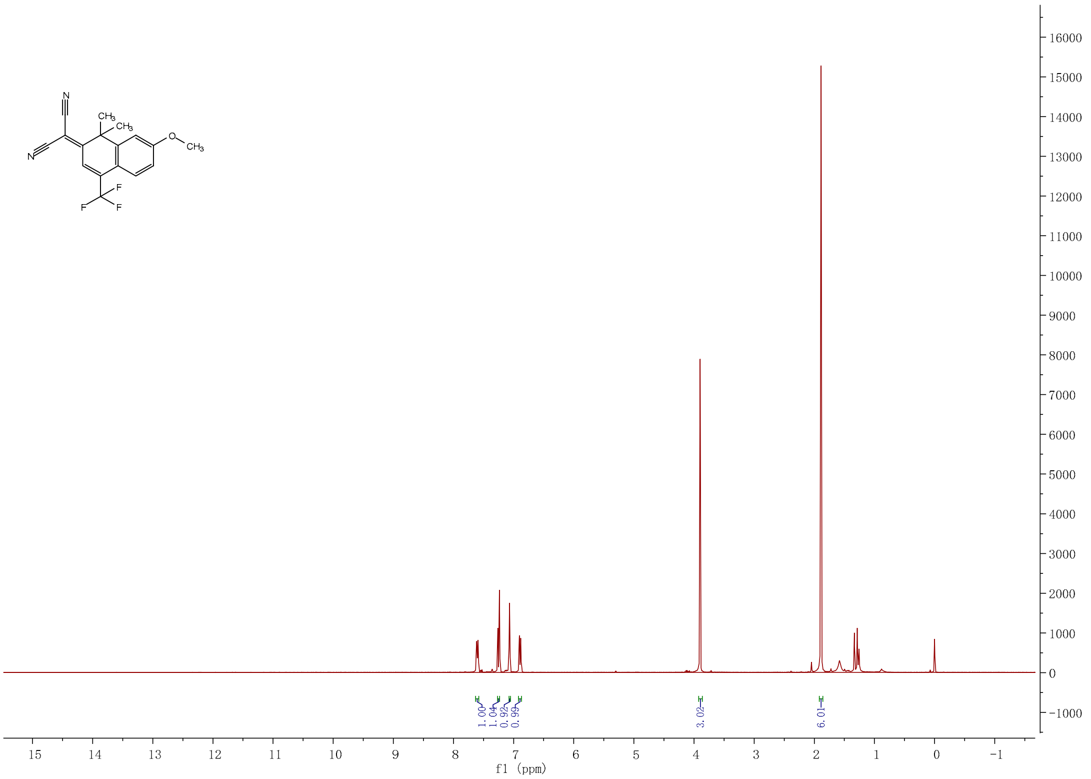

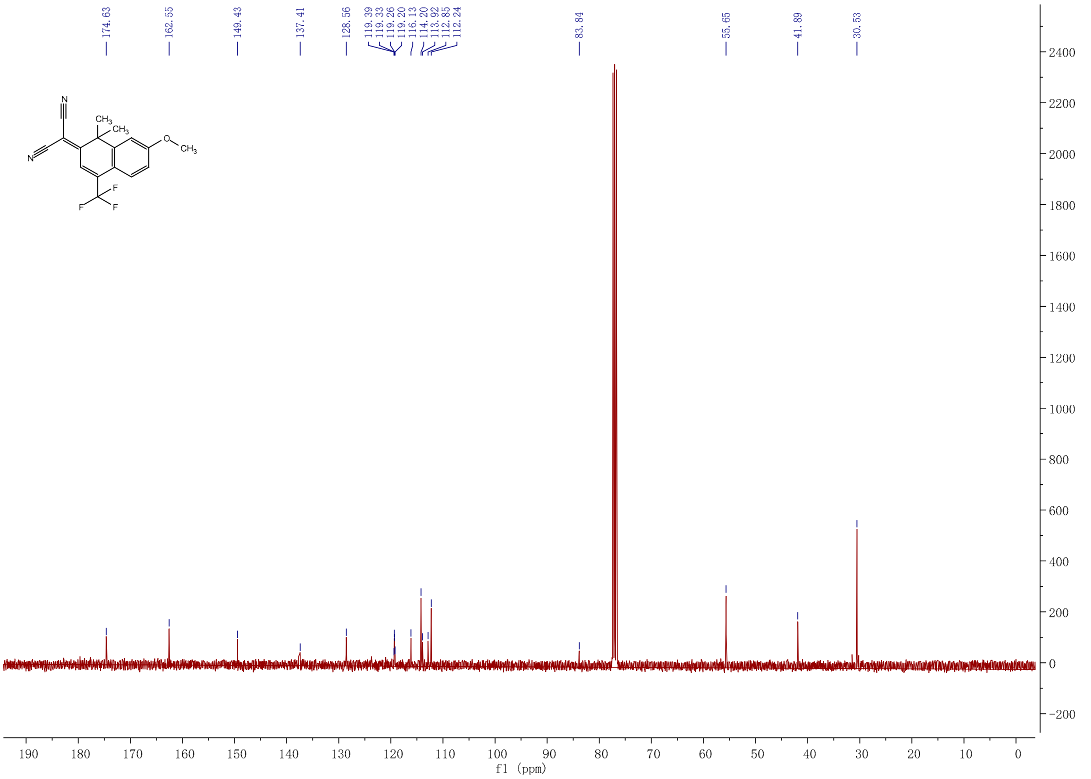

NC-4-F

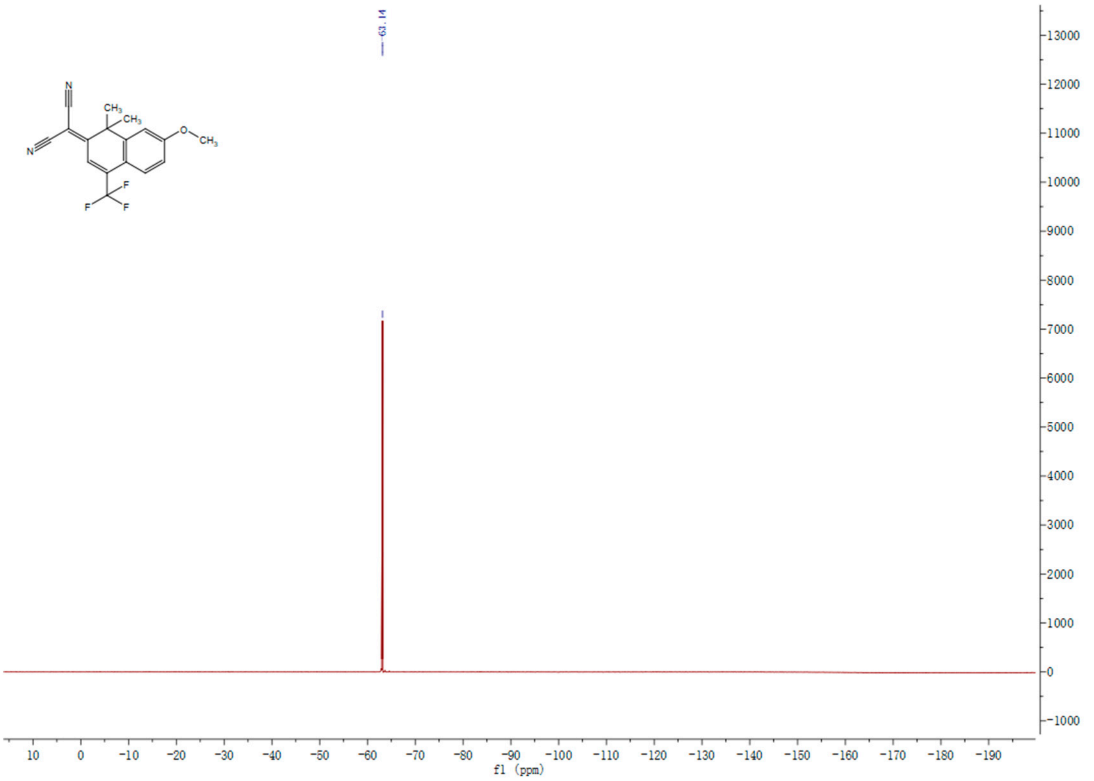

-63.14
13000
12000
11000
10000
9000
8000
7000
6000
5000
4000
3000
2000
1000
0
-1000
10
0
-10
-20
-30
-40
-50
-60
-70
-80
-90
-100
-110
-120
-130
-140
-150
-160
-170
-180
-190
f1 (ppm)

NC-4-Br

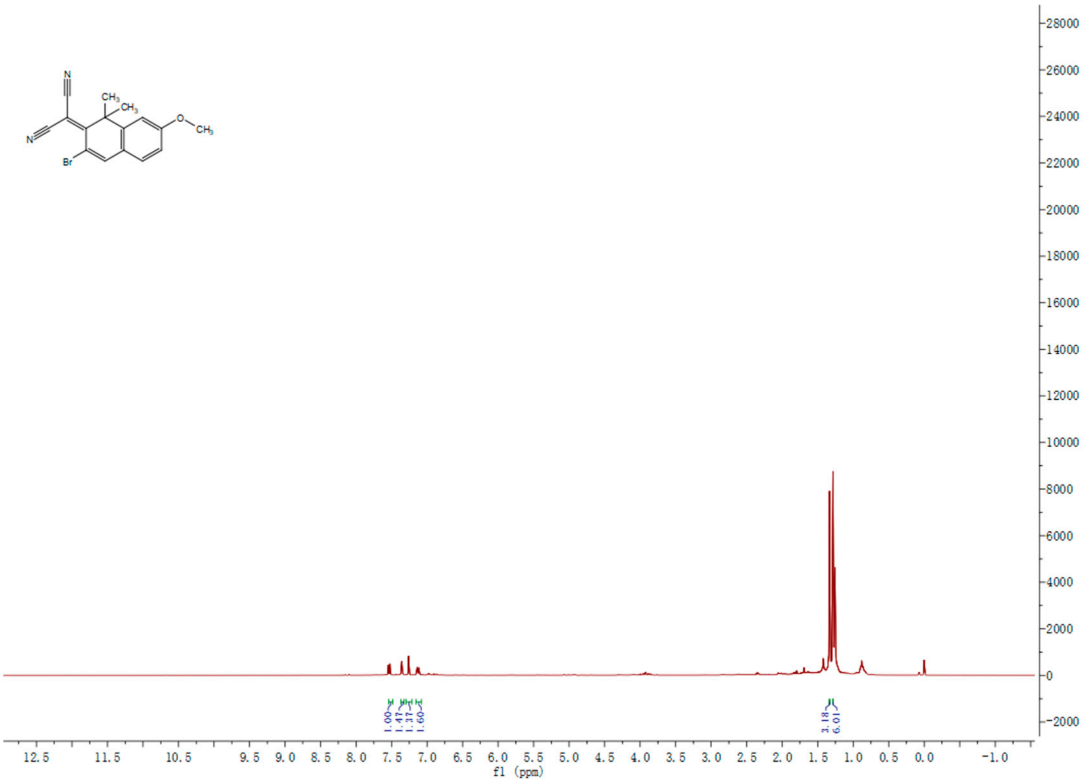

1.00
1.47
1.37
1.60
3.18
6.01
f1 (ppm)

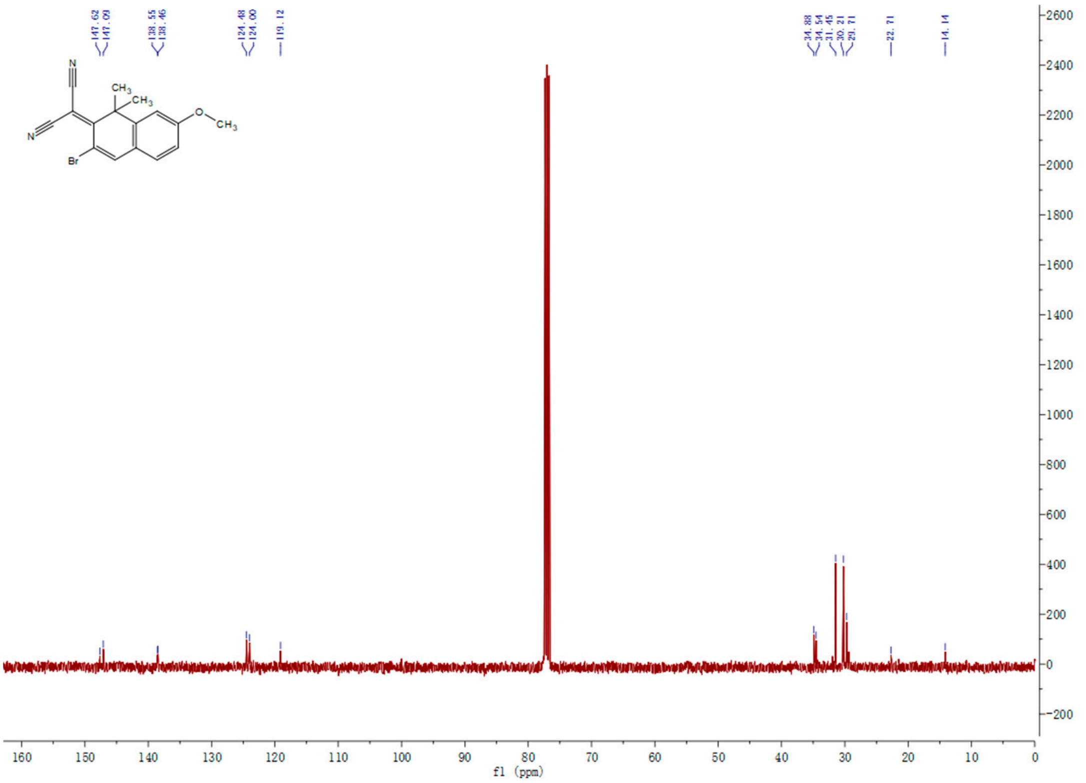

147.62
147.09
138.55
138.46
124.48
124.00
119.12
34.88
34.54
31.45
30.21
29.71
22.71
14.14
f1 (ppm)

NC-4-Ph

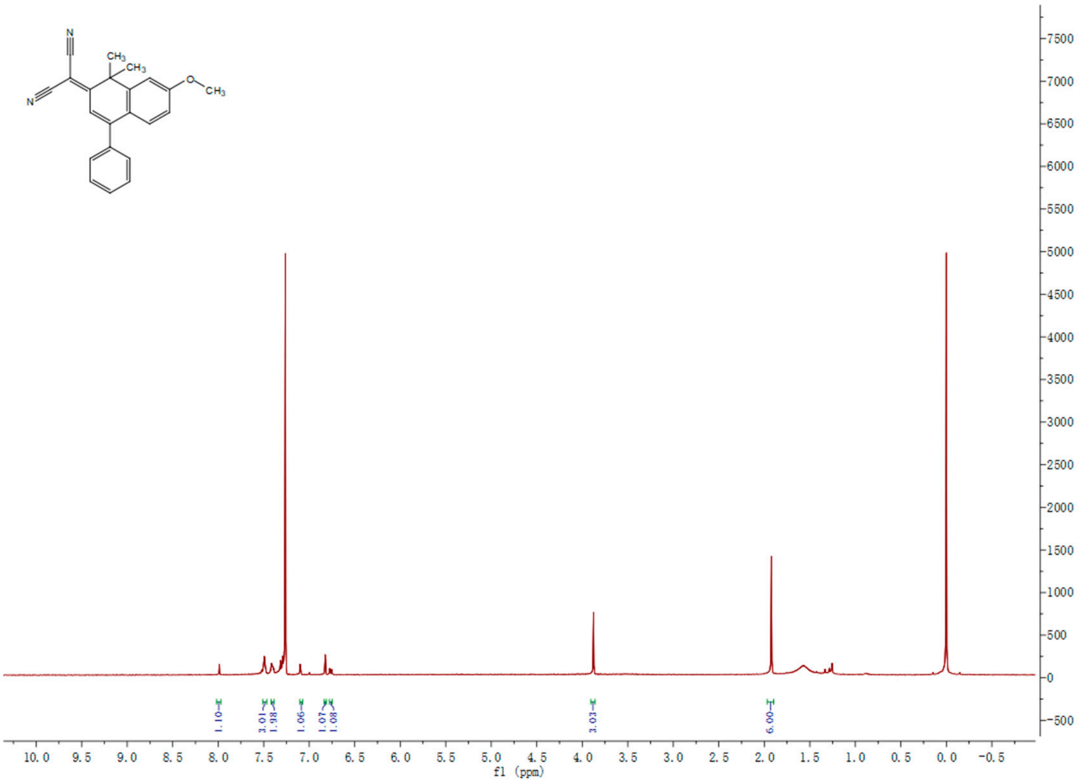

NC-5-IBX

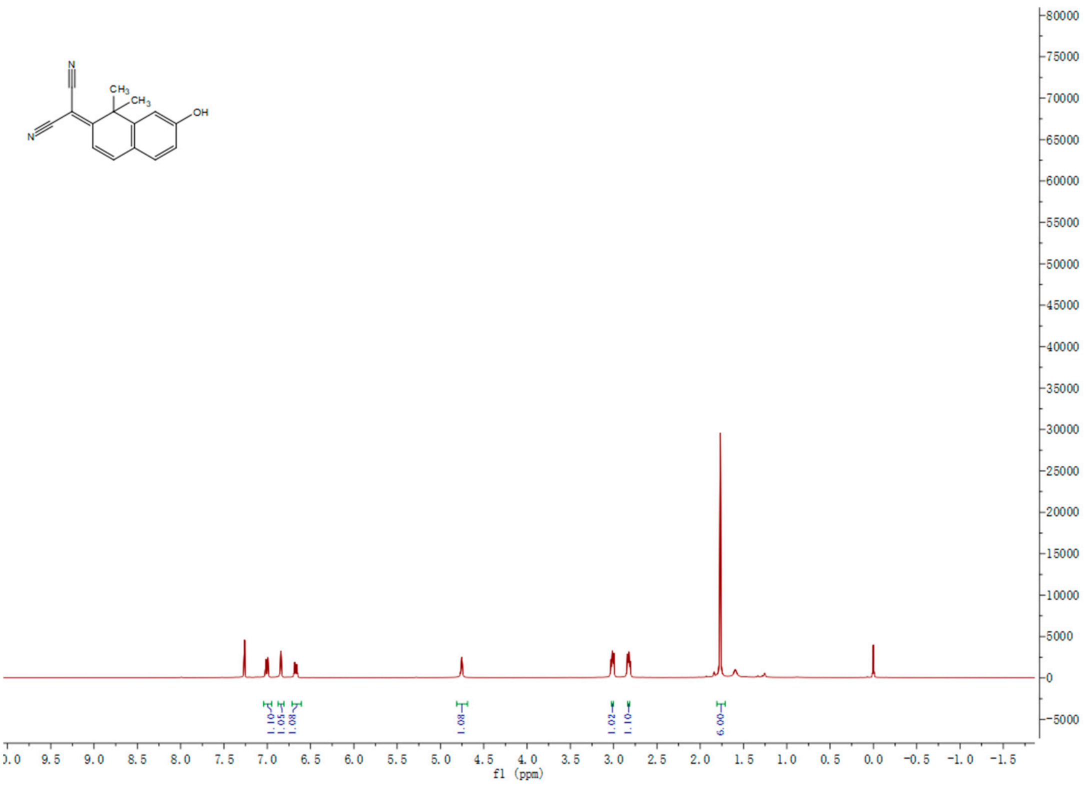

N
CH_3
CH_3
OH
N
1.10
1.05
1.08
1.08
1.02
1.10
6.00
f1 (ppm)

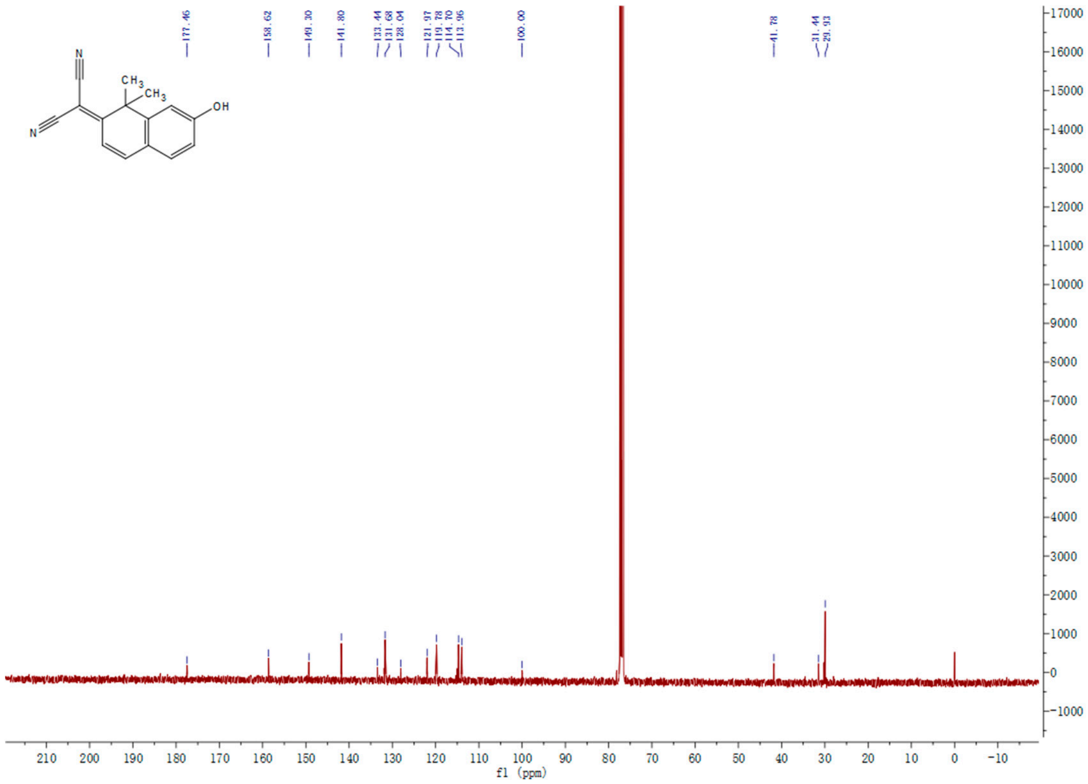

177.46
158.62
149.30
141.80
133.44
131.68
128.04
121.97
119.78
114.70
113.96
100.00
41.78
31.44
29.93
N
CH_3
CH_3
OH
N
f1 (ppm)

NC-5-3O

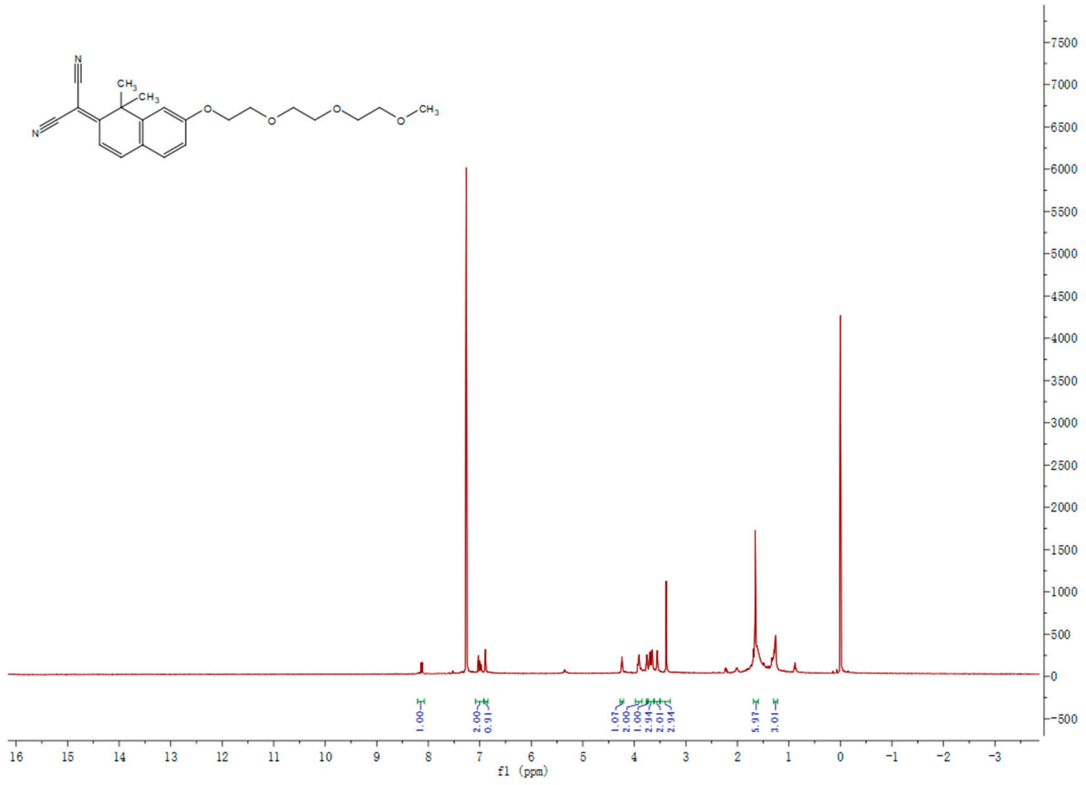

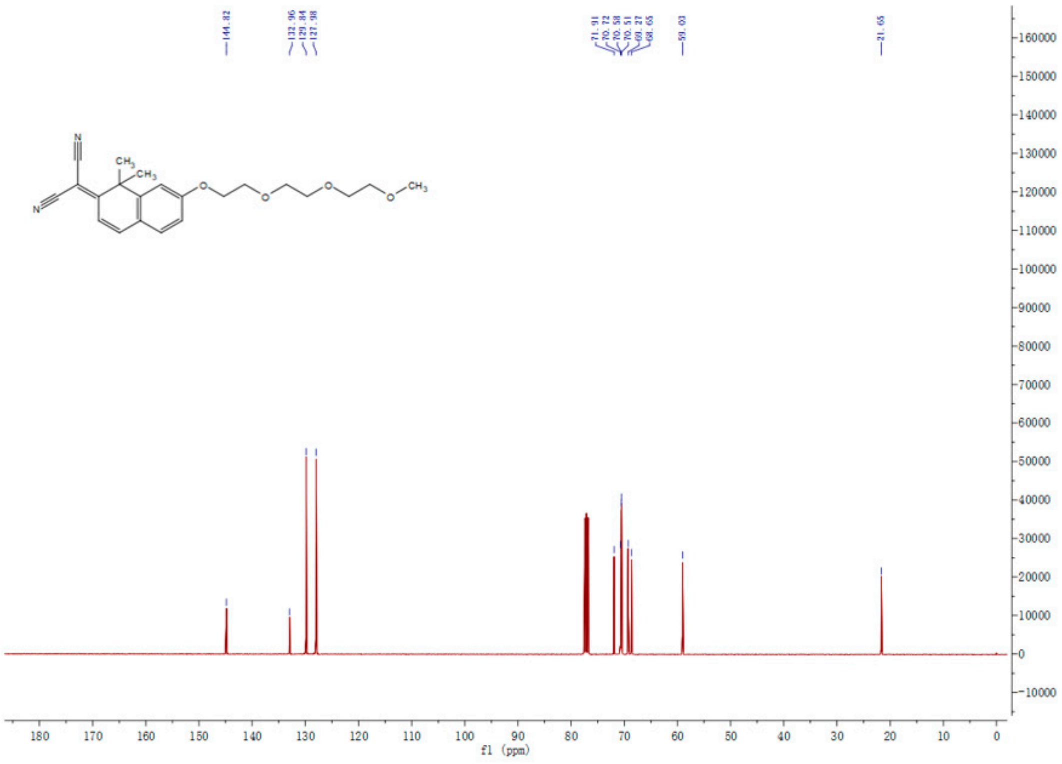

HPLC spectra

NC-4

Chromatogram and Results

Injection Details

Injection Name:	**NC-4-0421**	*Run Time (min):*	**25.00**
Vial Number:	**RB3**	*Injection Volume:*	**15.00**
Injection Type:	**Unknown**	*Channel:*	**UV_VIS_1**
Calibration Level:		*Wavelength:*	**254.0**
Instrument Method:	**ACN_5%_95%_400nm_25min**	*Bandwidth:*	**n.a.**
Processing Method:	**20160226**	*Dilution Factor:*	**1.0000**
Injection Date/Time:	**21/04/21 15:44**	*Sample Weight:*	**1.0000**

Chromatogram

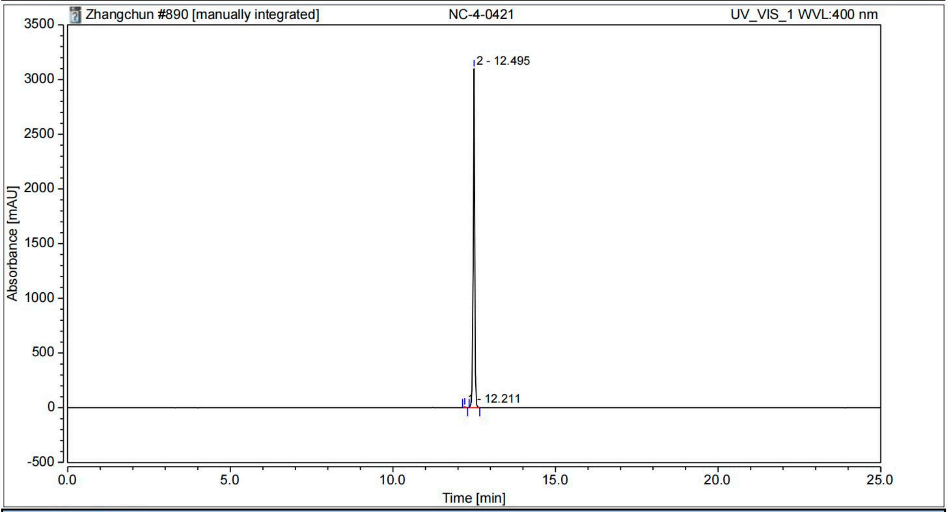

Integration Results

No.	Peak Name	Retention Time min	Area mAU*min	Height mAU	Relative Area %	Relative Height %	Amount n.a.
1		12.211	0.419	8.405	0.26	0.27	n.a.
2		12.495	161.439	3098.495	99.74	99.73	n.a.
**Total:**			**161.858**	**3106.900**	**100.00**	**100.00**	

NC-4-F

Chromatogram and Results

Injection Details

Injection Name:	**NC-4-F**	*Run Time (min):*	**25.00**
Vial Number:	**RB5**	*Injection Volume:*	**30.00**
Injection Type:	**Unknown**	*Channel:*	**UV_VIS_1**
Calibration Level:		*Wavelength:*	**400.0**
Instrument Method:	**ACN_5%_95%_254nm_25min-W**	*Bandwidth:*	**n.a.**
Processing Method:	**20160226**	*Dilution Factor:*	**1.0000**
Injection Date/Time:	**22/04/21 10:36**	*Sample Weight:*	**1.0000**

Chromatogram

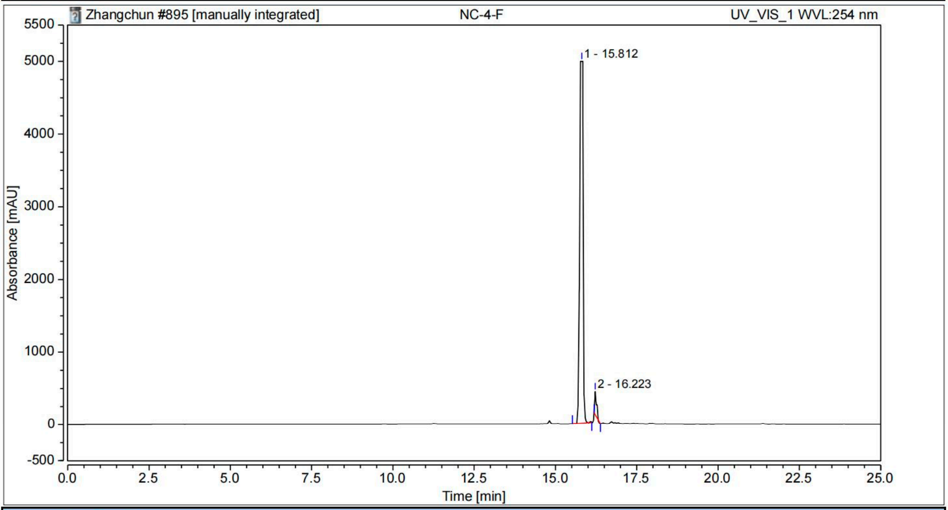

Integration Results

No.	Peak Name	Retention Time min	Area mAU*min	Height mAU	Relative Area %	Relative Height %	Amount
n.a.	Component 3	n.a.	n.a.	n.a.	n.a.	n.a.	n.a.
n.a.	Component 7	n.a.	n.a.	n.a.	n.a.	n.a.	n.a.
n.a.	Component 8	n.a.	n.a.	n.a.	n.a.	n.a.	n.a.
n.a.	Component 6	n.a.	n.a.	n.a.	n.a.	n.a.	n.a.
n.a.	Component 2	n.a.	n.a.	n.a.	n.a.	n.a.	n.a.
n.a.	Component 1	n.a.	n.a.	n.a.	n.a.	n.a.	n.a.
1		15.812	607.707	4978.874	96.89	94.03	n.a.
2		16.223	19.500	316.066	3.11	5.97	n.a.
n.a.	Component 4	n.a.	n.a.	n.a.	n.a.	n.a.	n.a.
n.a.	Component 5	n.a.	n.a.	n.a.	n.a.	n.a.	n.a.
**Total:**			**627.206**	**5294.940**	**100.00**	**100.00**	

NC-4-Br

Chromatogram and Results

Injection Details

Injection Name:	**NC-4-BR**	*Run Time (min):*	**25.00**
Vial Number:	**RA1**	*Injection Volume:*	**10.00**
Injection Type:	**Unknown**	*Channel:*	**UV_VIS_1**
Calibration Level:		*Wavelength:*	**254.0**
Instrument Method:	**ACN_5%_95%_254nm_25min-W -**	*Bandwidth:*	**n.a.**
Processing Method:	**20160226**	*Dilution Factor:*	**1.0000**
Injection Date/Time:	**19/03/22 10:11**	*Sample Weight:*	**1.0000**

Chromatogram

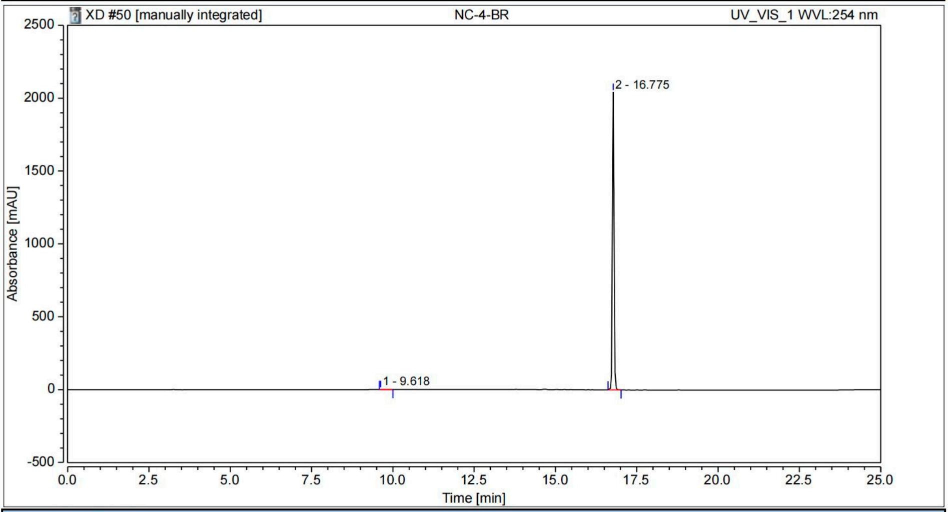

Integration Results

No.	Peak Name	Retention Time min	Area mAU*min	Height mAU	Relative Area %	Relative Height %	Amount n.a.
1		9.618	0.016	0.060	0.01	0.00	n.a.
2		16.775	118.049	2041.736	99.99	100.00	n.a.
**Total:**			**118.065**	**2041.795**	**100.00**	**100.00**	

NC-4-Ph

Chromatogram and Results

Injection Details

Injection Name:	**NC-4-PH**	*Run Time (min):*	**30.00**
Vial Number:	**RA1**	*Injection Volume:*	**50.00**
Injection Type:	**Unknown**	*Channel:*	**UV_VIS_1**
Calibration Level:		*Wavelength:*	**254.0**
Instrument Method:	**ACN_5%_95%_254nm_30min-20191228**	*Bandwidth:*	**n.a.**
Processing Method:	**20160226**	*Dilution Factor:*	**1.0000**
Injection Date/Time:	**16/07/21 14:02**	*Sample Weight:*	**1.0000**

Chromatogram

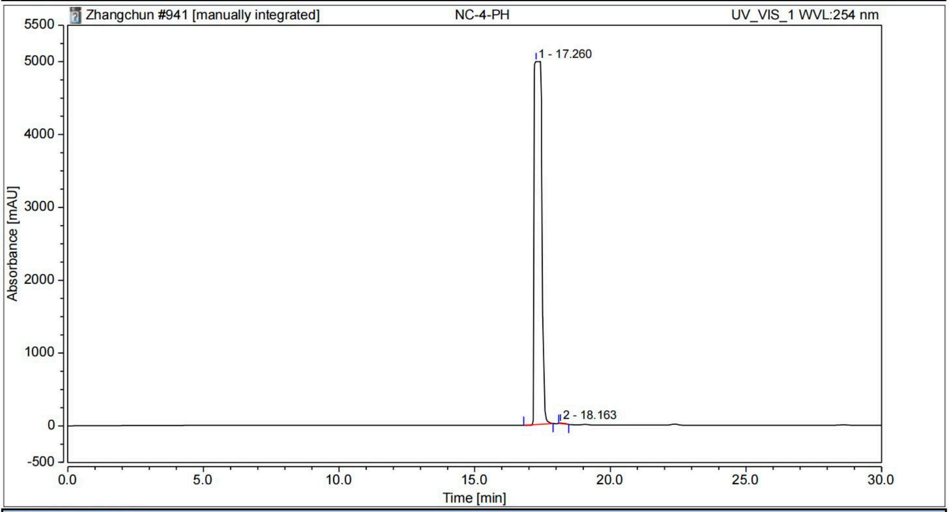

Integration Results

No.	Peak Name	Retention Time min	Area mAU*min	Height mAU	Relative Area %	Relative Height %	Amount
n.a.	Component 3	n.a.	n.a.	n.a.	n.a.	n.a.	n.a.
n.a.	Component 7	n.a.	n.a.	n.a.	n.a.	n.a.	n.a.
n.a.	Component 8	n.a.	n.a.	n.a.	n.a.	n.a.	n.a.
n.a.	Component 6	n.a.	n.a.	n.a.	n.a.	n.a.	n.a.
n.a.	Component 2	n.a.	n.a.	n.a.	n.a.	n.a.	n.a.
n.a.	Component 1	n.a.	n.a.	n.a.	n.a.	n.a.	n.a.
1		17.260	1533.765	4980.506	99.89	99.86	n.a.
2		18.163	1.667	6.939	0.11	0.14	n.a.
n.a.	Component 4	n.a.	n.a.	n.a.	n.a.	n.a.	n.a.
n.a.	Component 5	n.a.	n.a.	n.a.	n.a.	n.a.	n.a.
**Total:**			**1535.432**	**4987.445**	**100.00**	**100.00**	

NC-5-IBX

Chromatogram and Results

Injection Details

Injection Name:	**WST-NC-5-IBX**	*Run Time (min):*	**25.00**
Vial Number:	**RD1**	*Injection Volume:*	**10.00**
Injection Type:	**Unknown**	*Channel:*	**UV_VIS_1**
Calibration Level:		*Wavelength:*	**254.0**
Instrument Method:	**ACN_5%_95%_254nm_25min-W -**	*Bandwidth:*	**n.a.**
Processing Method:	**20160226**	*Dilution Factor:*	**1.0000**
Injection Date/Time:	**16/03/22 17:35**	*Sample Weight:*	**1.0000**

Chromatogram

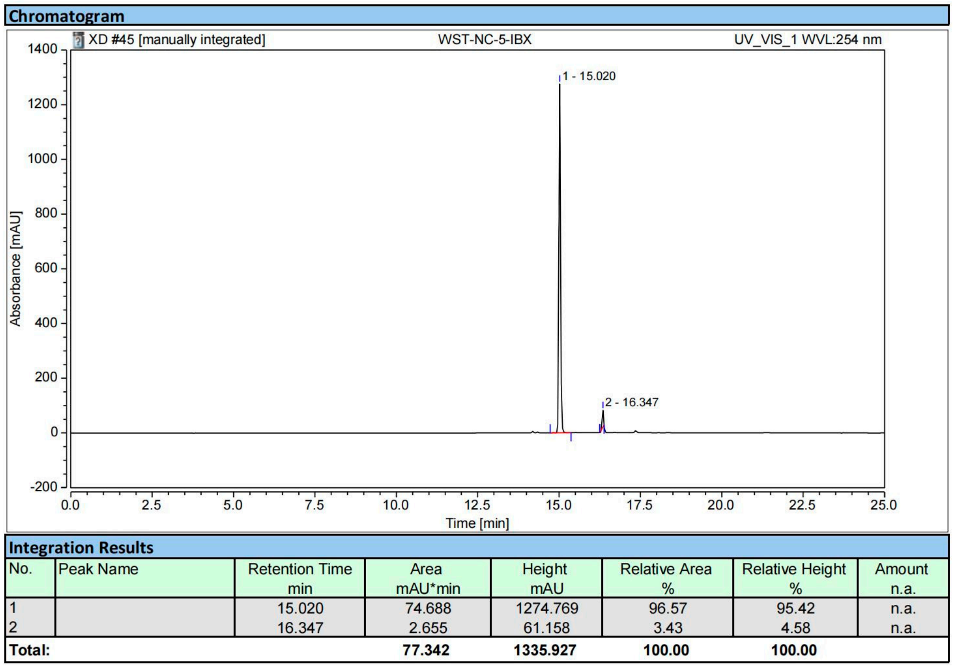

Integration Results

No.	Peak Name	Retention Time min	Area mAU*min	Height mAU	Relative Area %	Relative Height %	Amount n.a.
1		15.020	74.688	1274.769	96.57	95.42	n.a.
2		16.347	2.655	61.158	3.43	4.58	n.a.
**Total:**			**77.342**	**1335.927**	**100.00**	**100.00**	

NC-5-3O

Chromatogram and Results

Injection Details

Injection Name:	**NC-5-3O**	*Run Time (min):*	**30.00**
Vial Number:	**RA1**	*Injection Volume:*	**10.00**
Injection Type:	**Unknown**	*Channel:*	**UV_VIS_1**
Calibration Level:		*Wavelength:*	**254.0**
Instrument Method:	**ACN_5%_95%_254nm_30min-20191228**	*Bandwidth:*	**n.a.**
Processing Method:	**20160226**	*Dilution Factor:*	**1.0000**
Injection Date/Time:	**16/07/21 14:32**	*Sample Weight:*	**1.0000**

Chromatogram

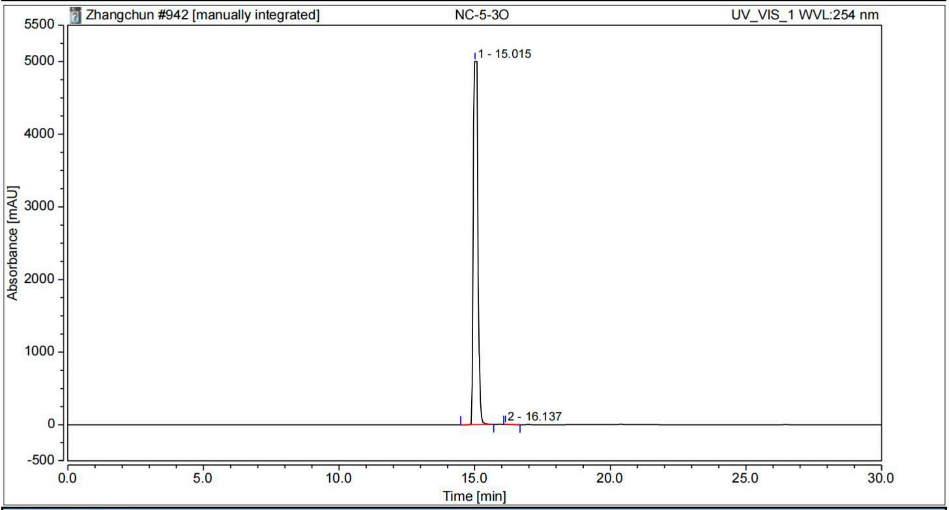

Integration Results

No.	Peak Name	Retention Time min	Area mAU*min	Height mAU	Relative Area %	Relative Height %	Amount
n.a.	Component 3	n.a.	n.a.	n.a.	n.a.	n.a.	n.a.
n.a.	Component 7	n.a.	n.a.	n.a.	n.a.	n.a.	n.a.
n.a.	Component 8	n.a.	n.a.	n.a.	n.a.	n.a.	n.a.
n.a.	Component 6	n.a.	n.a.	n.a.	n.a.	n.a.	n.a.
n.a.	Component 2	n.a.	n.a.	n.a.	n.a.	n.a.	n.a.
n.a.	Component 1	n.a.	n.a.	n.a.	n.a.	n.a.	n.a.
1		15.015	1001.865	4996.981	99.96	99.99	n.a.
2		16.137	0.379	0.480	0.04	0.01	n.a.
n.a.	Component 4	n.a.	n.a.	n.a.	n.a.	n.a.	n.a.
n.a.	Component 5	n.a.	n.a.	n.a.	n.a.	n.a.	n.a.
**Total:**			**1002.244**	**4997.461**	**100.00**	**100.00**	
